# Autophagy Signaling by Neural-Induced Human Adipose Tissue-Derived Stem Cell-Conditioned Medium during Rotenone-Induced Toxicity in SH-SY5Y Cells

**DOI:** 10.3390/ijms23084193

**Published:** 2022-04-10

**Authors:** Mahesh Ramalingam, Han-Seong Jeong, Jinsu Hwang, Hyong-Ho Cho, Byeong C. Kim, Eungpil Kim, Sujeong Jang

**Affiliations:** 1 Department of Physiology, Chonnam National University Medical School, Hwasun 58128, Korea; jhsjeong@hanmail.net (H.-S.J.); wlstn0128@naver.com (J.H.); 2 Department of Otolaryngology-Head and Neck Surgery, Chonnam National University Hospital, Chonnam National University Medical School, Gwangju 61469, Korea; victocho@jnu.ac.kr; 3 Department of Neurology, Chonnam National University Hospital, Chonnam National University Medical School, Gwangju 61469, Korea; byeong.kim7@gmail.com; 4 Jeonnam Biopharmaceutical Research Center, Hwasun 58141, Korea; keungpil@gmail.com

**Keywords:** Parkinson’s disease, LC3B, rotenone, mTOR, mesenchymal stem cells

## Abstract

Rotenone (ROT) inhibits mitochondrial complex I, leading to reactive oxygen species formation, which causes neurodegeneration and alpha-synuclein (α-syn) aggregation and, consequently, Parkinson’s disease. We previously found that a neurogenic differentiated human adipose tissue-derived stem cell-conditioned medium (NI-hADSC-CM) was protective against ROT-induced toxicity in SH-SY5Y cells. In the present study, ROT significantly decreased the phospho (p)-mTORC1/total (t)-mTOR, p-mTORC2/t-mTOR, and p-/t-ULK1 ratios and the ATG13 level by increasing the DEPTOR level and p-/t-AMPK ratio. Moreover, ROT increased the p-/t-Akt ratio and glycogen synthase kinase-3β (GSK3β) activity by decreasing the p-/t-ERK1/2 ratios and beclin-1 level. ROT also promoted the lipidation of LC3B-I to LC3B-II by inducing autophagosome formation in Triton X-100-soluble and -insoluble cell lysate fractions. Additionally, the levels of ATG3, 5, 7, and 12 were decreased, along with those of lysosomal LAMP1, LAMP2, and TFEB, leading to lysosomal dysfunction. However, NI-hADSC-CM treatment increased the p-mTORC1, p-mTORC2, p-ULK1, p-Akt, p-ERK1/2, ATG13, and beclin-1 levels and decreased the p-AMPK level and GSK3β activity in response to ROT-induced toxicity. Additionally, NI-hADSC-CM restored the LC3B-I level, increased the p62 level, and normalized the ATG and lysosomal protein amounts to control levels. Autophagy array revealed that the secreted proteins in NI-hADSC-CM could be crucial in the neuroprotection. Taken together, our results showed that the neuroprotective effects of NI-hADSC-CM on the autophagy signaling pathways could alleviate the aggregation of α-syn in Parkinson’s disease and other neurodegenerative disorders.

## 1. Introduction

Parkinson’s disease (PD), a chronic neurodegenerative disorder (NDD) that progresses with age and greatly impacts the quality of life of afflicted patients, is characterized by motor and nonmotor symptoms that are caused by dopaminergic neuronal defects in the substantia nigra of the midbrain. Oxidative stress, mitochondrial dysfunction, the aggregation of misfolded proteins, and autophagy dysfunction are involved in the pathogenesis of PD [1]. It has been suggested that intracellular aggregation and accumulation of alpha-synuclein (α-syn) of various forms (including monomeric, dimeric, oligomeric, and fibrillary forms) in Lewy bodies and Lewy neurites expressed in the presynaptic structures of the brain are responsible for the disease [2]. The accumulation of α-syn oligomers at the presynaptic membrane induces mitochondrial dysfunction and promotes oxidative stress-mediated toxicity, apoptosis, and cell death and, thereby, the pathogenesis of PD [3]. Our previous research findings suggested that insoluble aggregated α-syn oligomers could be the primary source of the toxicity in PD, whereas soluble α-syn oligomers might not be pathogenic [4]. Under normal circumstances, macroautophagy (autophagy)-related processes interact with aggregated α-syn oligomers for their processing and elimination in the degradation pathways to main intracellular protein homeostasis [5]. During autophagy, long-lived proteins, excess or damaged organelles, and aggregated proteins are engulfed within a double-membrane vesicle to form autophagosomes, which then fuse with lysosomes to form autolysosomes. The proteins sequestered in these compartments are subsequently degraded by various lysosomal hydrolases, generating amino acids that are recycled for macromolecular synthesis and energy production [6]. Therefore, defects in the protein degradation system during autophagy would lead to the enhanced deposition of α-syn aggregates in Lewy bodies [7,8].

Mechanistic target of rapamycin (mTOR), a major negative regulator of autophagy, is a serine (Ser)/threonine (Thr) kinase that regulates various physiological processes, including cell growth, proliferation, and survival [9]. mTOR forms two complexes, mTORC1 and mTORC2 [10], which have different substrate specificities [11]. The activation and inactivation of mTOR signaling are involved in the different stages of PD pathogenesis [12]. DEP domain-containing mTOR-interacting protein (DEPTOR) is an mTOR-binding protein that inhibits both mTORC1 and mTORC2 endogenously [13]. AMP-activated protein kinase (AMPK) is another major nutrient- and cell energy-sensing protein that responds to oxidative stress in NDDs. The AMPK and mTOR pathways directly regulate Unc-51-like kinase 1 (ULK1) at Ser757 [1,14]. ULK1 interacts with autophagy-related protein 13 (ATG13), forming a complex that is a critical initiator of autophagy [12] to integrate the pathogenic processes of PD.

Protein kinase B (PKB/Akt), another serine/threonine kinase, is a strong survival signal that blocks intrinsic and extrinsic apoptosis [15]. Glycogen synthase kinase-3 beta (GSK3β), a ubiquitously expressed serine/threonine kinase and one of the substrates of Akt, has been shown to be directly involved in neurotoxic cell death mediated by the PI3K–mTOR pathway [16]. Akt inhibits GSK3β activity by phosphorylating it at Ser9 [17]. It was reported that the three-dimensional structure of GSK3 mostly resembles that of mitogen-activated protein kinase (MAPK) [16]. Extracellular signal-regulated kinases (ERK1/2), which have been implicated in protein synthesis, also phosphorylate GSK3β for its inactivation [18]. Additionally, beclin-1, an ortholog of vacuolar protein sorting-associated protein 30 (VPS30, also known as ATG6), is important for the localization of autophagic proteins to the preautophagosomal structure regulated by ULK1 [19] and ERK [20] and is involved in several critical cellular processes during autophagy.

Autophagy is a continuous process that is executed by various ATGs. In NDDs, the clearance of abnormal proteins is conducted by the ubiquitin proteasome system and/or autophagy–lysosome system. The incorporation of microtubule-associated protein 1 light chain 3 beta (MAP1LC3B; also known as ATG8 or LC3B) into Lewy bodies [21] suggests that the autophagy–lysosome pathway is involved in the pathogenesis of PD [22]. The formation of LC3B-II (14 kDa; a specific marker of phagophores and autophagosomes) from its cytosolic precursor LC3B-I is related to the maturation of autophagosomes [23]. Sequestosome-1 (SQSTM1 or p62), an autophagy substrate, is a long-lived scaffolding protein that facilitates the transport of ubiquitinated proteins [24] by binding to LC3-like proteins within autophagolysosomes [25] for successful removal of insoluble proteins [26]. The failure to degrade Lewy bodies in autophagolysosomes as a result of lysosomal degradation dysfunction has been demonstrated to be a cause of the neurodegeneration in NDDs [23].

Rotenone (ROT) is a lipophilic piscicide that is derived from the roots of plants belonging to the genera *Lonchocarpus* and *Derris* [4]. It crosses the blood–brain barrier, accumulates in subcellular organelles, and suppresses the flow of electrons from the iron–sulfur centers in mitochondrial respiratory chain complex I, leading to the formation of reactive oxygen species (ROS), which can induce oxidative stress in the cells [27]. ROT was noted for its ability to cause selective nigrostriatal dopaminergic degeneration and induce the formation of α-syn aggregates and the ensuing cognitive and motor deficits [28]. In our previous study, ROT-induced toxicity increased the phosphorylation of α-syn and its aggregation in SH-SY5Y neuroblastoma cells, which induced their apoptotic death [4]. Therefore, we surmised that the ROT-induced neurotoxicity in SH-SY5Y cells could be used as a model to gain insights into the pathophysiological mechanisms of PD.

In our exploration of potential therapeutic strategies for PD, we recently evaluated the protective effects of neural-induced (NI) human adipose tissue-derived stem cell (hADSC)-conditioned medium (NI-hADSC-CM) against ROT-induced PD-like impairments [4]. The mesenchymal stem cells used for the therapy were self-renewing, and their beneficial effects against NDDs were found to be due mainly to their secretomes in models of PD [29,30]. hADSCs differentiate into neuronal-like cells (NI-hADSCs) in the presence of basic fibroblast growth factor (bFGF) and forskolin for over 2 weeks [31]. Additionally, NI-hADSC-CM treatment reduced SH-SY5Y apoptosis by decreasing the phosphorylation of α-syn molecules and their aggregation [4]. To develop a deeper understanding of the mechanisms underlying the therapeutic effects of NI-hADSC-CM, this study was carried out to investigate the role of this medium in α-syn degradation by various autophagy-related pathways.

## 2. Results

### 2.1. Effects of NI-hADSC-CM on ROT-Induced ROS Formation in SH-SY5Y Cells

According to our previous studies, 0.5 μM of ROT could reduce cell viability to 55% relative to the untreated control group and was therefore used as the concentration in this study. Additionally, NI-hADSC-CM at 50% dilution showed therapeutic effects against the ROT-induced decrease in cell viability and was thus also used in these subsequent experiments [4]. A schematic of the experimental study plan is depicted in Supplementary Figure S1b.

**Figure 1:**
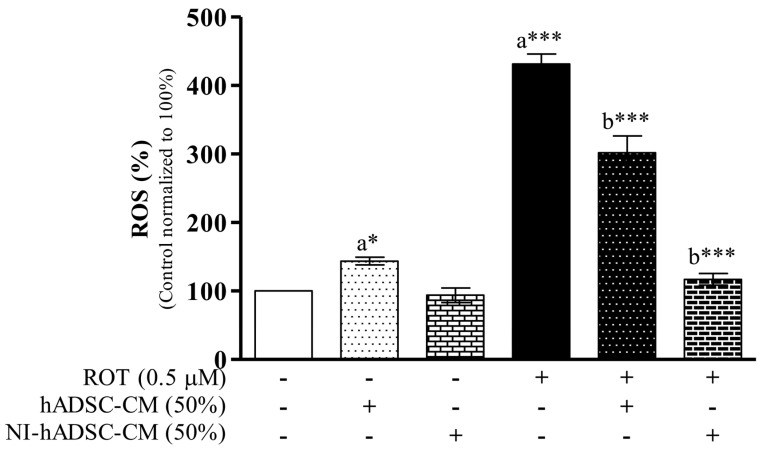
Effect of NI-hADSC-CM on ROT-induced ROS generation. SH-SY5Y cells were seeded at a density of 50,000 cells/mL in DMEM containing 1% FBS and used for experiments after overnight incubation. Cells were incubated in the absence or presence of ROT (0.5 μM) for 48 h and then treated with hADSC-CM or NI-hADSC-CM (at 50% dilution each) during the last 24 h. ROS was assessed using the H_2_DCFDA assay. Data are presented as the mean ± SEM of three independent experiments. Statistical analysis was performed using one-way analysis of variance followed by Tukey’s post hoc test. Statistical significance: a—compared with control; b—compared with ROT; * *p* < 0.05 and *** *p* < 0.001.

In this study, the level of ROS generation was measured to determine the inhibitory effect of ROT on mitochondrial complex I after 48 h. As shown in Figure 1, ROS production was significantly higher in the ROT-exposed cells (*p* < 0.001) than in the control cells not exposed to the piscicide, suggesting that the ROT-induced toxicity could lead to apoptosis-mediated neurodegeneration in SH-SY5Y cells, which was reported in our previous study [4]. However, treatment with NI-hADSC-CM strongly inhibited the ROT-promoted formation of ROS (*p* < 0.001), indicating that the therapy could provide protection against ROT-induced toxicity. By contrast, treatment of the control cells with hADSC-CM significantly increased their ROS level (*p* < 0.05). These results revealed that NI-hADSC-CM had higher therapeutic effects than hADSC-CM in SH-SY5Y cells.

### 2.2. Effects of NI-hADSC-CM on mTORC1, mTORC2, and DEPTOR Protein Expression in ROT-Exposed SH-SY5Y Cells

**Figure 2:**
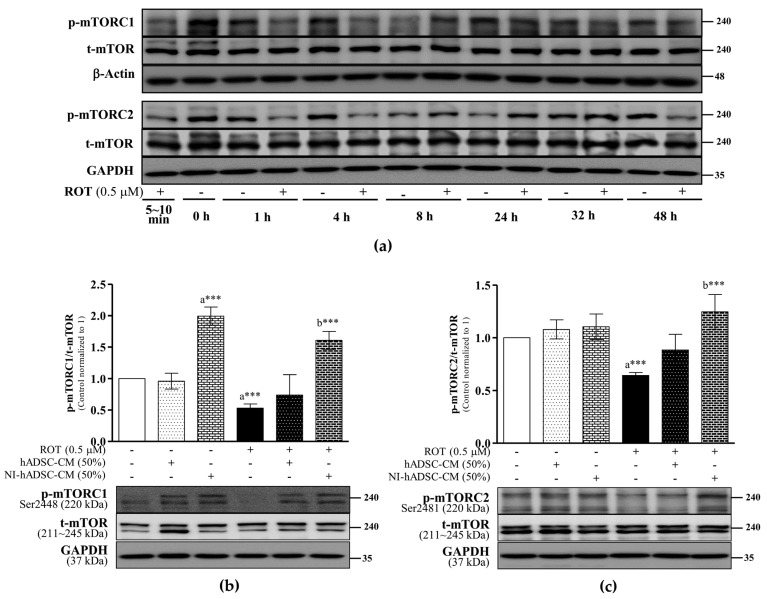
Effect of NI-hADSC-CM on mTOR regulation. SH-SY5Y cells were treated for different timepoints with or without ROT (0.5 μM) and assessed by Western blotting (**a**). SH-SY5Y cells were incubated in the absence or presence of ROT (0.5 μM) for 48 h and treated with hADSC-CM or NI-hADSC-CM (at 50% dilution each) during the last 24 h. The ratios of p-mTORC1/t-mTOR (**b**) and p-mTORC2/t-mTOR (**c**) proteins were assessed using the Western blot assay. Original uncut Western blot images are shown in Supplementary Figure S3. Data are presented as the mean ± SEM of three independent experiments. Statistical analysis was performed using one-way analysis of variance followed by Tukey’s post hoc test. Statistical significance: a—compared with control; b—compared with ROT; *** *p* < 0.001. p-, phosphorylated; t-, total.

The post-translational modification of mTOR is a key step in the regulation of autophagy, with mTORC1 and mTORC2 undergoing phosphorylation at Ser2448 and Ser2481, respectively [1]. Using a time-course study, the changes in expression levels of the mTOR complexes were monitored in SH-SY5Y cells exposed to ROT for 0–48 h. (A schematic of the experimental plan is depicted in Supplementary Figure S1a.) The toxicity induced by ROT significantly decreased (*p* < 0.001) the phosphorylation of mTORC1-Ser2448 (Figure 2a,b) at 24 h and 48 h. mTORC2-Ser2481 (Figure 2a,c) activity was increased at 24 h but decreased at 48 h, leading to neuronal dysfunction. By contrast, NI-hADSC-CM treatment upregulated the phosphorylation of both mTORC1 (Figure 2b) and mTORC2 (Figure 2c) in the ROT-exposed cells (*p* < 0.001). However, treatments with ROT, hADSC-CM, or NI-hADSC-CM alone did not change the total levels of mTORC1 and mTORC2. Moreover, hADSC-CM treatment did not have any significant effects on the levels of the phosphorylated complexes in the ROT-exposed or control cells (Figure 2b,c).

### 2.3. Effects of NI-hADSC-CM on AMPK, ULK1, and ATG13 Protein Expression in ROT-Exposed SH-SY5Y Cells

**Figure 3:**
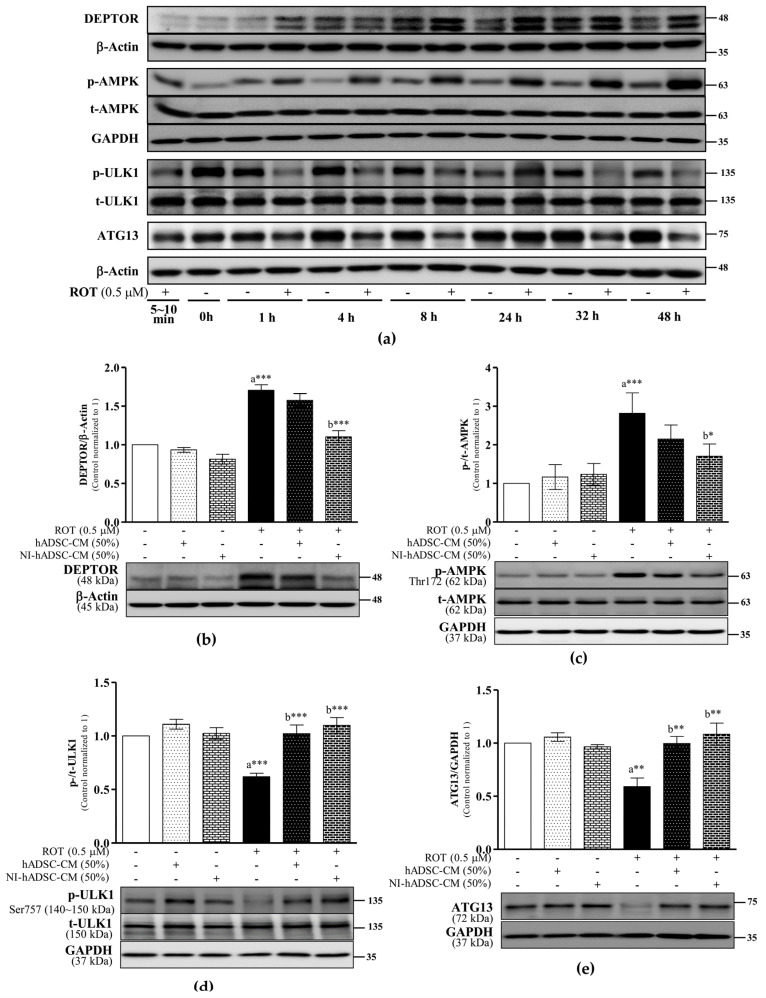
Effects of NI-hADSC-CM on DEPTOR, AMPK, and ULK1/ATG13 complex signaling. SH-SY5Y cells were treated for different timepoints with or without ROT (0.5 μM) and assessed by Western blotting (**a**). SH-SY5Y cells were incubated in the absence or presence of ROT (0.5 μM) for 48 h and then treated with hADSC-CM or NI-hADSC-CM (at 50% dilution each) during the last 24 h. The levels of DEPTOR (**b**), p-/t-AMPK (**c**), p-/t-ULK1 (**d**), and ATG13 (**e**) protein expression were assessed using the Western blot assay. Original uncut Western blot images are shown in Supplementary Figure S4. Data are presented as the mean ± SEM of three independent experiments. Statistical analysis was performed using one-way analysis of variance followed by Tukey’s post hoc test. Statistical significance: a—compared with control; b—compared with ROT; * *p* < 0.05, ** *p* < 0.01, and *** *p* < 0.001. p-, phosphorylated; t-, total.

DEPTOR, a component of both mTORC1 and mTORC2, usually contributes to the deregulation of mTOR signaling. The DEPTOR protein level was significantly increased (*p* < 0.001) at 24 h and 48 h in response to the ROT-induced toxicity in the SH-SY5Y cells (Figure 3a,b). By contrast, NI-hADSC-CM treatment significantly prevented the ROT-induced increase in DEPTOR protein expression, as evidenced by Western blot results (*p* < 0.001; Figure 3b). Taken together, these findings confirm that hADSC-CM has no protective effect against ROT-induced toxicity and indicate that NI-hADSC-CM prevents mTOR dysfunction, allowing the activation of mTORC1 and mTORC2 to eventually lead to SH-SY5Y cell survival.

mTOR is a downstream target of AMPK, which is activated by phosphorylation at Thr172 [32]. The initiation of autophagy is induced by the interactions of AMPK with the Unc-51-like kinase 1 (ULK1) and ATG13 complex. In the ROT-exposed SH-SY5Y cells, AMPK was activated by its phosphorylation (*p* < 0.001; Figure 3a,c) at 24 h and 48 h. The ULK1 phosphorylation level and ATG13 level were increased at 24 h by ROT, which in turn inhibited ULK1 phosphorylation (*p* < 0.001; Figure 3a,d) and decreased the ATG13 level (*p* < 0.01; Figure 3a,e) at 48 h. By contrast, NI-hADSC-CM treatment significantly reduced the activation of AMPK (Thr172) (*p* < 0.05), thereby leading to higher ULK1 (Ser757) phosphorylation (*p* < 0.001) and ATG13 levels (*p* < 0.01). Although hADSC-CM significantly increased the levels of p-ULK1 (*p* < 0.001) and ATG13 (*p* < 0.01) in the ROT-exposed cells, it had no effect on AMPK (*p* > 0.05). Moreover, neither hADSC-CM nor NI-hADSC-CM had any effects on these proteins in the control cells. These results suggest that NI-hADSC-CM can inhibit AMPK and DEPTOR expression and increase mTOR and ULK1/ATG13 complex signaling in ROT-exposed cells.

### 2.4. Effects of NI-hADSC-CM on Akt, ERK, GSK3β, and Beclin-1 Protein Expression in ROT-Exposed SH-SY5Y Cells

**Figure 4:**
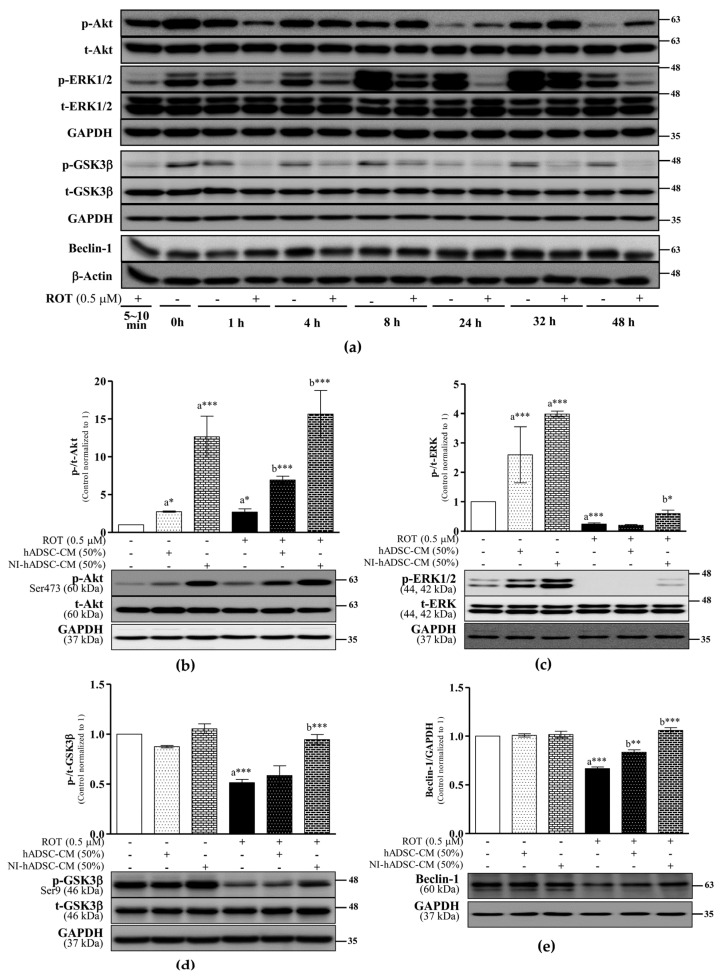
Effects of NI-hADSC-CM on Akt and ERK signaling. SH-SY5Y cells were treated for different timepoints with or without ROT (0.5 μM) and assessed by Western blotting (**a**). SH-SY5Y cells were incubated in the absence or presence of ROT (0.5 μM) for 48 h and then treated with hADSC-CM or NI-hADSC-CM (at 50% dilution each) during the last 24 h. The levels of p-/t-Akt (**b**), p-/t-ERK1/2 (**c**), p-/t-GSK3β (**d**), and beclin-1 (**e**) protein expression were assessed using the Western blot assay. Original uncut Western blot images are shown in Supplementary Figure S5. Data are presented as the mean ± SEM of three independent experiments. Statistical analysis was performed using one-way analysis of variance followed by Tukey’s post hoc test. Statistical significance: a—compared with control; b—compared with ROT; * *p* < 0.05, ** *p* < 0.01, and *** *p* < 0.001. p-, phosphorylated; t-, total.

The Akt (PKB) and ERK1/2 signaling pathways promote cell survival by phosphorylating several downstream targets, thereby blocking intrinsic and extrinsic apoptosis. As a downstream target of Akt and ERK1/2, GSK3β is inactivated through its phosphorylation at Ser9 by these kinases [33]. In this study, the ROT-induced toxicity activated Akt phosphorylation at Ser473 (*p* < 0.05; Figure 4a,b), decreased the phosphorylation of ERK1/2 at Thr202/tyrosine 204 (*p* < 0.001; Figure 4a,c), and dephosphorylated GSK3β-Ser9 for its activation (*p* < 0.001; Figure 4a,d) at 48 h.

The phosphorylation of Akt in the ROT-exposed cells was further increased (*p* < 0.001) by treatments with either hADSC-CM or NI-hADSC-CM. Moreover, NI-hADSC-CM noticeably augmented the phosphorylation of ERK1/2 (*p* < 0.05), subsequently leading to the phosphorylation of GSK3β (*p* < 0.001), which reduced its activity to normal levels. The hADSC-CM treatment did not have any effects on ERK1/2 or GSK3β during ROT-induced toxicity. In the control cells, hADSC-CM and NI-hADSC-CM significantly increased the p-Akt (*p* < 0.05 and *p* < 0.001, respectively) and p-ERK1/2 levels (*p* < 0.001, both). These results suggest that NI-hADSC-CM can significantly reduce ROT-induced apoptosis and promote cell survival.

Beclin-1 (ATG6) is important for the localization of autophagic proteins to the preautophagosomal structure regulated by ULK1 and ERK and is involved in several critical cellular processes during autophagy [19,20]. In this study, the ROT-induced toxicity significantly decreased the beclin-1 level at 48 h (*p* < 0.001; Figure 4a,e), but this effect was significantly reversed with the hADSC-CM and NI-hADSC-CM treatments (*p* < 0.01 and *p* < 0.001, respectively). These results suggest that ROT-induced toxicity is involved in the initiation of autophagy and that this effect can be ameliorated by NI-hADSC-CM.

### 2.5. Effects of NI-hADSC-CM on LC3B and p62 Protein Expression in ROT-Exposed SH-SY5Y Cells

Autophagy is a multistage process known as the autophagic flux. A study of LC3B (ATG8) revealed that the number of autophagosomes increased in proportion to the band intensity of phosphatidylethanolamine-conjugated LC3B-II, which migrated faster than LC3B-I when electrophoresed on sodium dodecyl sulfate (SDS)-polyacrylamide gels [34]. p62 links aggregated or ubiquitinated proteins to the autophagic process through interaction with LC3B.

**Figure 5:**
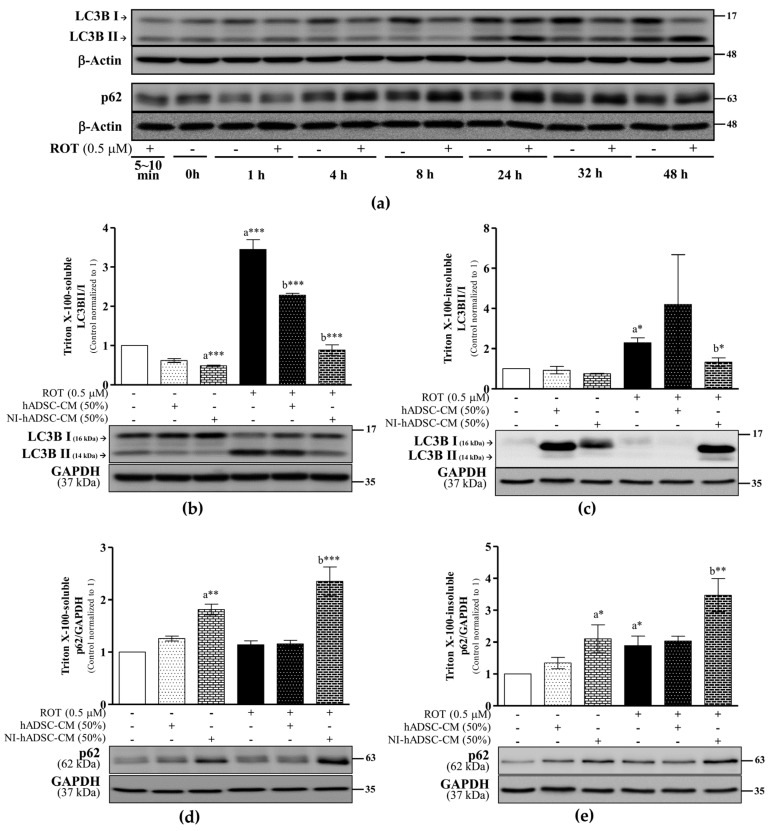
Effects of NI-hADSC-CM on LC3B and p62 protein expressions. SH-SY5Y cells were treated for different timepoints with or without ROT (0.5 μM) and Triton X-100-soluble cell lysate fractions assessed by Western blotting (**a**). SH-SY5Y cells were incubated in the absence or presence of ROT (0.5 μM) for 48 h and then treated with hADSC-CM or NI-hADSC-CM (at 50% dilution each) during the last 24 h. The levels of LC3B (**b**,**c**) and p62 (**d**,**e**) proteins in Triton X-100-soluble (**b**,**d**) and Triton X-100-insoluble (**c**,**e**) cell lysate fractions were assessed using the Western blot assay. Original uncut Western blot images are shown in Supplementary Figure S6. Data are presented as the mean ± SEM of three independent experiments. Statistical analysis was performed using one-way analysis of variance followed by Tukey’s post hoc test. Statistical significance: a—compared with control; b—compared with ROT; * *p* < 0.05, ** *p* < 0.01, and *** *p* < 0.001.

In our time-course study, ROT increased the ratio of LC3B-II/LC3B-I at 48 h in the Triton X-100-soluble cell lysate fraction. The LC3B-II levels were also increased at 24 and 48 h of ROT-induced toxicity compared with their levels in the respective time-based control groups (Figure 5a). Moreover, although the p62 level was significantly higher in the ROT-exposed cells than in the control cells at 24 h, there was no difference in the levels between the two cell groups at 48 h (Figure 5a). These results showed that ROT-induced toxicity had inhibited the autophagic flux at 24 h despite inducing the formation of autophagosomes, and the levels of the autophagy-related molecules were saturated at 48 h (Figure 5a). To verify the role of NI-hADSC-CM in this process, Western blot analysis of the autophagy markers in Triton X-100-soluble and -insoluble cell lysate fractions was carried out. As expected, ROT-induced toxicity significantly increased the ratio of LC3B-II/LC3B-I in the soluble (*p* < 0.001; Figure 5b) and insoluble (*p* < 0.05; Figure 5c) fractions, whereas treatment with NI-hADSC-CM significantly decreased the ROT effects in both fractions (soluble: *p* < 0.001, Figure 5b; and insoluble: *p* < 0.05, Figure 5c). However, hADSC-CM treatment significantly decreased the LC3B-II/LC3B-I ratio in the soluble fraction only in the ROT-exposed cells (*p* < 0.001; Figure 5b). Additionally, NI-hADSC-CM treatment also decreased the ratio of LC3B-II/LC3B-I in the soluble fraction of control cells (*p* < 0.001; Figure 5b). These results suggest that NI-hADSC-CM regulates autophagosome formation not only during ROT-induced toxicity but also in the absence of the piscicide.

The amount of p62 protein is inversely proportional to the magnitude of autophagy. We found that the toxicity induced by ROT for 48 h significantly increased the p62 level in the insoluble cell lysate fraction only compared with that in the control sample. The increased LC3B-II/LC3B-I ratio and p62 level in the insoluble fraction were indications of ROT-induced impairment of the autophagic flux and lysosomal inhibition. However, quantitative analysis of the soluble (Figure 5d) and insoluble (Figure 5e) fractions showed that NI-hADSC-CM treatment increased the levels of p62 in both the ROT-exposed (soluble: *p* < 0.001 and insoluble: *p* < 0.01) and control cells (*p* < 0.01 and *p* < 0.05, respectively). Taken together, these results suggest that ROT induces the autophagic flux with soluble proteins but inhibits the autophagy–lysosomal flux with insoluble aggregated proteins. Importantly, the reduced or impaired autophagic flux that occurs during NI-hADSC-CM treatment suggests the role of this treatment in mTOR signaling-related cell survival.

### 2.6. Effects of NI-hADSC-CM on ATG Protein Expression in ROT-Exposed SH-SY5Y Cells

**Figure 6:**
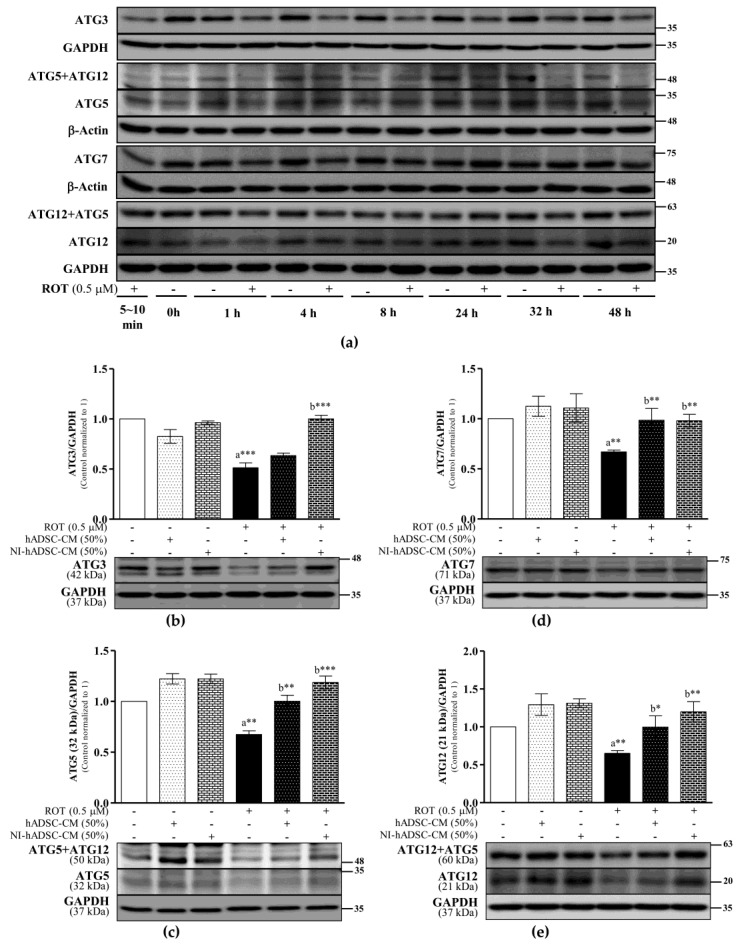
Effects of NI-hADSC-CM on ATG protein expressions. SH-SY5Y cells were treated for different timepoints with or without ROT (0.5 μM) and assessed by Western blotting (**a**). SH-SY5Y cells were incubated in the absence or presence of ROT (0.5 μM) for 48 h and then treated with hADSC-CM or NI-hADSC-CM (at 50% dilution each) during the last 24 h. The levels of ATG3 (**b**), ATG5 (**c**), ATG7 (**d**), and ATG12 (**e**) protein expression were assessed using the Western blot assay. Original uncut Western blot images are shown in Supplementary Figure S7. Data are presented as the mean ± SEM of three independent experiments. Statistical analysis was performed using one-way analysis of variance followed by Tukey’s post hoc test. Statistical significance: a—compared with control; b—compared with ROT; * *p* < 0.05, ** *p* < 0.01, and *** *p* < 0.001.

Autophagy is regulated by the ATG family of proteins, which regulate the formation and maturation of the autophagosomes [35]. In this study, the toxicity induced by ROT decreased the expression levels of ATG3 (*p* < 0.001; Figure 6b), ATG5 (*p* < 0.01; Figure 6c), ATG7 (*p* < 0.01; Figure 6d), and ATG12 (*p* < 0.01; Figure 6e) significantly over time. The conjugation of ATG5–ATG12 (*p* < 0.01; Supplementary Figure S2a) and of ATG12–ATG5 (*p* < 0.001; Supplementary Figure S2b) also decreased in the ROT-exposed SH-SY5Y cells. Importantly, NI-hADSC-CM treatment reversed the ROT-induced effects by significantly stimulating the expression levels of ATG3 (*p* < 0.001), ATG5 (*p* < 0.001), ATG7 (*p* < 0.01), and ATG12 (*p* < 0.01) and increasing the conjugation of ATG5–ATG12 (*p* < 0.01; Supplementary Figure S2a) and of ATG12–ATG5 (*p* < 0.001; Supplementary Figure S2b). hADSC-CM also significantly increased the ATG5 (*p* < 0.01), ATG7 (*p* < 0.01), and ATG12 (*p* < 0.05) levels and ATG12–ATG5 conjugation (*p* < 0.05; Supplementary Figure S2b) in the ROT-treated cells. The decrease in the ATG levels in the ROT-exposed cells suggests that the increased formation of autophagosomes consequently increased autophagy and apoptosis, all of which NI-hADSC-CM suppressed and thereby protected the SH-SY5Y cells.

### 2.7. Effects of NI-hADSC-CM on Lysosomal Protein Expression in ROT-Exposed SH-SY5Y Cells

Once autophagosomes are formed, they are shuttled to lysosomes for fusion and subsequent degradation [35]. Autophagosome–lysosome fusion is facilitated by lysosomal-associated membrane protein-1 (LAMP1), LAMP2, and transcription factor EB (TFEB).

**Figure 7:**
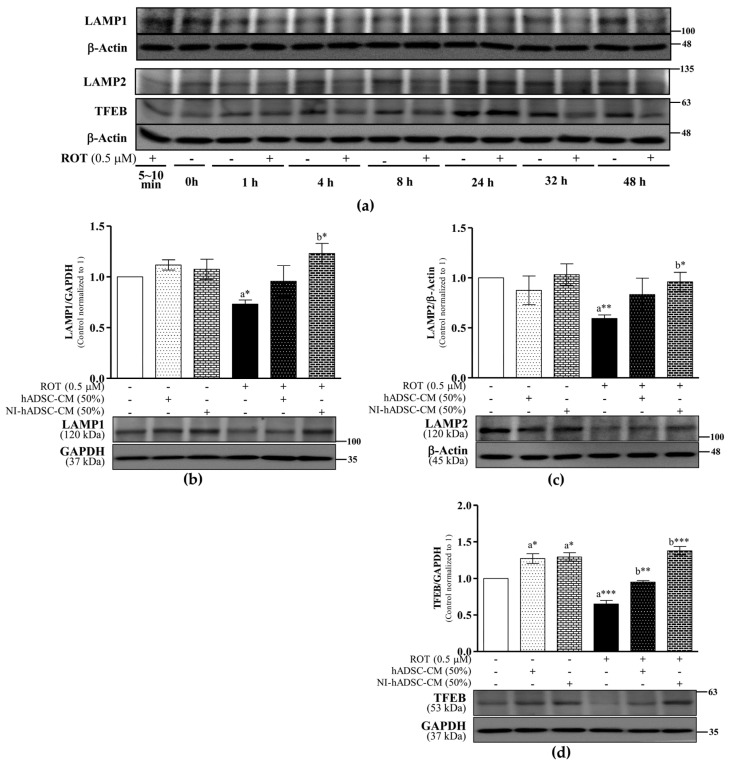
Effects of NI-hADSC-CM on lysosomal protein expressions. SH-SY5Y cells were treated for different timepoints with or without ROT (0.5 μM) and assessed by Western blotting (**a**). SH-SY5Y cells were incubated in the absence or presence of ROT (0.5 μM) for 48 h and then treated with hADSC-CM or NI-hADSC-CM (at 50% dilution each) during the last 24 h. The levels of LAMP1 (**b**), LAMP2 (**c**), and TFEB (**d**) protein expression were assessed using the Western blot assay. Original uncut Western blot images are shown in Supplementary Figure S8. Data are presented as the mean ± SEM of three independent experiments. Statistical analysis was performed using one-way analysis of variance followed by Tukey’s post hoc test. Statistical significance: a—compared with control; b—compared with ROT; * *p* < 0.05, ** *p* < 0.01, and *** *p* < 0.001.

In the SH-SY5Y cells exposed to ROT for 48 h, the LAMP1 (*p* < 0.05; Figure 7b), LAMP2 (*p* < 0.01; Figure 7c), and TFEB levels (*p* < 0.001; Figure 7d) were significantly decreased relative to the levels in the control cells. By contrast, NI-hADSC-CM treatment significantly upregulated the levels of these lysosomal proteins (LAMP1 and LAMP2: *p* < 0.05; TFEB: *p* < 0.001). Additionally, hADSC-CM treatment increased the TFEB levels in the ROT-exposed cells. Moreover, the overexpression of TFEB (*p* < 0.05) was also found in the control SH-SY5Y cells treated with hADSC-CM and NI-hADSC-CM. These results suggest that ROT impairs autophagosome degradation, an effect that NI-hADSC-CM ameliorates, thereby contributing to dopaminergic neuronal regeneration.

### 2.8. Analysis of Autophagy Antibody Array in Conditioned Mediums

**Figure 8:**
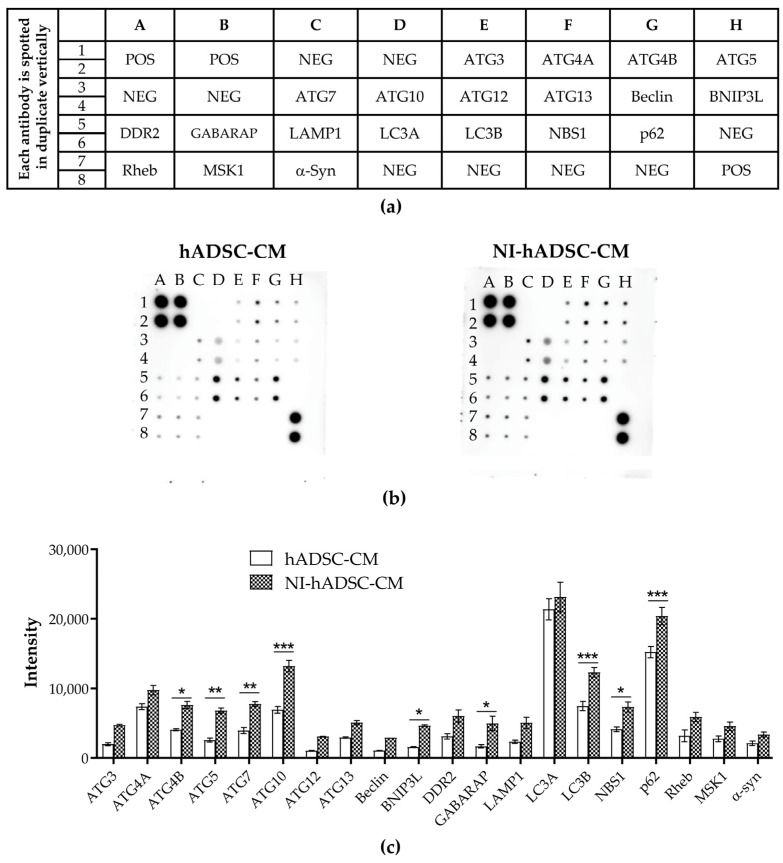
Proteomic membrane analysis of stem cell-conditioned mediums. (**a**) The human autophagy array (Raybiotech, AAH-ATG-1-8) profiles 20 autophagy-related proteins spotted in duplicate, positive controls (POS), and negative controls (NEG). (**b**) Collected hADSC-CM and NI-hADSC-CM were examined with the use of an antibody-based protein array assay. Results were evaluated after exposure of the array membranes for 10 min. Original uncut image is shown in Supplementary Figure S9. (**c**) Quantification of the 20 proteins related to autophagy. Data are presented as the mean ± SEM. Statistical analysis was performed using two-way analysis of variance. Statistical significance: NI-hADSC-CM compared with hADSC-CM as control; * *p* < 0.05, ** *p* < 0.01, and *** *p* < 0.001.

In order to identify the secreted components of the NI-hADSC-CM that triggered the above effects, the autophagy-related proteins were evaluated in the hADSC-CM and NI-hADSC-CM by an autophagy array assay (Figure 8). Based on the obtained protein profiles (Supplementary Table S2), ATG3, ATG4A, ATG4B, ATG5, ATG7, ATG10, ATG12, ATG13, Beclin-1, BNIP3L, DDR2, GABARAP, LAMP1, LC3B, NBS1, p62, Rheb, MSK1, and α-syn were expressed (>20%), and a level of LC3A was secreted (8%). In addition, ATG4B, ATG5, ATG7, ATG10, BNIP3L, GABARAP, LC3B, NBS1, and p62 results were statistically higher in the NI-hADSC-CM than in the hADSC-CM (Figure 8c; Supplementary Table S2).

## 3. Discussion

Stem cell-based therapies have been proposed for the treatment of NDDs, where cultured human mesenchymal stem cells differentiated into neuronal cells have significant advantages over primary stem cells. We had previously reported that culture medium containing a low percentage of fetal bovine serum (FBS) and supplemented with bFGF and forskolin can facilitate the differentiation of primary hADSC into neuron-like NI-hADSCs that express elevated levels of neural marker genes and proteins [31,36,37]. Their secretome or conditioned medium constitutes a complex mixture of soluble products, including lipids, proteins, nucleic acids, chemokines, cytokines, growth factors, hormones, microvesicles, and exosomes [38,39], which are believed to be modulators of several biological processes and could thus provide promising therapeutic effects against ROT-induced toxicity [4,40].

As demonstrated in our previous study, the exposure of SH-SY5Y cells to 0.5 μM of ROT for 48 h significantly reduced their viability, but the addition of NI-hADSC-CM (50% dilution in DMEM + 1% FBS) in the last 24 h of the 48 h ROT exposure reversed the toxic effect and increased the cell number [4]. In this present study, ROT induced an increase in the ROS levels, which was confirmed by the decrease in SH-SY5Y cell viability. ROT inhibits mitochondrial complex I, which increases the forward electron flow and reduces cellular oxygen into radicals, leading to excess ROS formation and cell death by oxidative stress, with dopaminergic neuronal dysfunction ensuing [41,42]. The prevention of ROS formation by NI-hADSC-CM treatment provides evidence that ROT-induced cell death is likely mediated by the inhibition of complex I and the production of ROS. Moreover, the effects of hADSC-CM in increasing ROS formation in the control SH-SY5Y cells and decreasing their viability (the latter demonstrated in our previous study [4]) suggest that this particular conditioned medium might not be a suitable therapeutic agent for NDDs; therefore, the effects of hADSC-CM on various cellular signaling events determined in this study are not discussed herein.

The ROT-induced toxicity toward SH-SY5Y cells causes many of the molecular dysfunctions associated with PD [4,40]. We previously also reported an increase in α-syn phosphorylation and observed significantly increased levels of α-syn oligomers in ROT-exposed SH-SY5Y cells [4]. The interactions of α-syn with the ubiquitin, lysosomal, and chaperone proteins prevent the rapid detection of aggregated α-syn and its processing or elimination [43]. The results of this study suggest that there may be a failure of the cellular protein degradation system during ROT-induced toxicity [44]. Inhibition of the oxidative stress induced by aggregated α-syn accumulation is essential for treating PD [45]. As a major α-syn degradation pathway, autophagy contributes toward this goal of inhibiting α-syn aggregation [46,47]. Therefore, autophagy dysfunction would enhance the deposition of α-syn aggregates [7,8], promoting cellular oxidative stress and death [48]. Indeed, increased levels of α-syn and ROS and the impairment of autophagy during ROT-induced toxicity have been reported [49]. Given these finding, the elucidation of the cellular and molecular mechanisms of various signaling pathways involved in autophagy would lead to a better understanding of the pathogenesis of PD. The master regulator of autophagy is mTOR, which modulates the process negatively [44].

mTOR signaling, which acts as a regulator of cellular growth and functions, is also involved in neural development, neuronal functions, and autophagy [11]. Imbalances in the mTOR signaling pathway are strongly associated with NDDs, such as Alzheimer’s disease and PD. mTOR is composed of mTORC1 (p-Ser2448) and mTORC2 (p-Ser2481), both of which have shared and distinct components and can be regulated by phosphorylation [11,49,50]. In this study, ROT inhibited the phosphorylation of both mTORC1 and mTORC2, resulting in apoptosis and the inhibition of cell growth, as also reported previously [51]. Moreover, the combined inhibition of mTORC1 and mTORC2 was suggested as an approach to block the feedback compensation that follows the therapeutic blockade of mTORC1 [52]. DEPTOR (DEPDC6), an endogenous inhibitor of mTORC1 and mTORC2, acts by binding to the mTOR FAT domain in both complexes [13]. In this study, the ROT-induced increase in the DEPTOR level was alleviated by NI-hADSC-CM treatment. As previously shown, growth factors can promote the phosphorylation of mTORC1 and mTORC2, and the proper activation of mTORs enhances dopaminergic neuronal survival [50,51], which is crucial to PD prevention. Moreover, a decrease in the DEPTOR level was previously shown to lead to increases in both mTORC1 and mTORC2 activities in neuroblastoma cells [53].

AMPK is a well-known metabolic energy sensor [45]. It has been reported that ROS, calcium, and the AMP/ATP ratio can regulate AMPK activation through its phosphorylation, which also plays a role in autophagy and cell energy homeostasis [54]. The finding in this study that ROT increased the phosphorylation of AMPK at Thr172 in SH-SY5Y cells is supported by the results of another study, in which ROT activated AMPK and thereby contributed to the suppression of mTOR and the induction of neuronal cell death [55]. The direct inhibition of mTORC1 mediated through AMPK activation for the phosphorylation of raptor [32] has been reported to induce autophagy [56]. Interestingly, NI-hADSC-CM treatment inhibited AMPK signaling by lowering its phosphorylation and significantly attenuated the cell death mediated by ROT-induced apoptotic and autophagic signaling.

Moreover, autophagy induction depends on the activity of ULK1 (ATG1), which interacts with ATG13 to promote autophagosome formation [57]. ULK1, a gatekeeper for autophagy initiation, is a key downstream molecule of the mTORC1 signaling pathway [50]. In this study, ROT activated AMPK and suppressed the mTOR complexes, which resulted in the dephosphorylation of ULK1 at Ser757. Treatment with NI-hADCSC-CM increased the levels of ULK1 phosphorylation and ATG13, suggesting that mTOR inhibits autophagy through the activation of ULK1 [58]. Additionally, ULK1 has the ability to autophosphorylate with ATG13 [59]. These results show that ROT causes cell death by initiating autophagy and that NI-hADSC-CM treatment inhibits autophagy-associated apoptosis by regulating the mTOR/AMPK/ULK1 signaling complex in SH-SY5Y cells.

Akt and MAPK are important signaling enzymes in the transduction of various signals. As a downstream signaling molecule of mTOR, Akt plays a central role in promoting cell growth, cell survival, apoptosis, and autophagy in PD [50]. Akt and mTORC1/2 are linked to one another through positive and negative regulatory feedback mechanisms [60]. Akt activation requires its phosphorylation at Ser473 [53]. Our finding of the increased phosphorylation of Akt at Ser437 in the ROT-exposed cells suggests the ability of this enzyme to attenuate the toxicity of the piscicide. A previous study also showed that oxidative stress activated the Akt survival signaling pathway [61,62]. NI-hADSC-CM treatment also enhanced the p-/t-Akt ratio in response to the ROT-induced toxicity in SH-SY5Y cells. The increased mTOR and decreased DEPTOR levels can lead to the phosphorylation of Akt-Ser473 to regulate cell survival, metabolism, and cytoskeletal organization [10,63]. Additionally, Akt phosphorylation triggers mTORC1 activation and contributes to the suppression of autophagy [64].

The Akt signaling pathway also regulates the phosphorylation of several downstream targets [65], such as GSK3, a ubiquitously expressed serine/threonine protein kinase that phosphorylates and inactivates glycogen synthase. GSK3 is involved in nutrient sensing, gene transcription, mitochondrial function, autophagy, and apoptosis [16]. ROT-induced activation of the isozyme GSK3β, which regulates neuronal survival, has been implicated in the pathogenesis of PD [66]. Studies have shown that α-syn can recruit and activate GSK3β [67,68], which exerts proapoptotic activity to induce autophagic cell death [16]. In this study, we also found that ROT increased GSK3β activity through its dephosphorylation at Ser9 with increased α-syn aggregation. By contrast, NI-hADSC-CM treatment deactivated GSK3β by phosphorylating Ser9. Numerous kinases, such as Akt and ERK, have been shown to inactivate GSK3β by phosphorylating it at Ser9 [69], thereby suppressing cytodestructive autophagy in neurons [70]. We also previously reported [4] that an increased level of Akt upregulated myeloid cell leukemia-1 (Mcl-1), a B-cell lymphoma-2 (Bcl-2) family member, via the inhibition of GSK3 [71].

ERKs reside within the microtubular network where their activities are associated with microtubules, memory, and synaptic plasticity [72,73]. In this study, the decreased phosphorylation of ERK1/2 suggests intracellular crosstalk between the mTOR and ERK1/2 signaling pathways, as was previously reported [74]. As we and others have previously reported, activation of the ERK1/2 and mTOR signaling pathways by NI-hADSC-CM appears to bring about the upregulated expression of proteins related to neuronal differentiation, such as neuron-specific class III β-tubulin (Tuj1) in neuroblastoma cells [4,53,75]. Because DEPTOR contains a putative ERK-binding site, its inhibition results in increased ERK1/2 phosphorylation independently of the mTOR pathway [76]. Taken together, these results suggest that the NI-hADSC-CM-mediated activation of Akt and ERK is associated with cell-protective effects elicited through the inhibition of oxidative stress and apoptosis.

Beclin-1 (ATG6), a multifunctional protein that binds to the antiapoptotic protein Bcl-2, is an important regulator of autophagy initiation (as a phagophore inducer), being involved in the recruitment of membranes to form autophagosomes for regulating cell death [77,78]. Beclin-1 interacts with several binding partners and can both induce and suppress the autophagy pathway [79]. In this study, ROT-induced toxicity decreased the beclin-1 level in SH-SY5Y cells. Such decrease in beclin-1 levels has been known to promote abnormal lysosomal functions associated with insufficient autophagy clearance [78,80]. The depletion of beclin-1 triggers caspase-dependent programmed cell death [20], which was also proven in our previous study [4]. NI-hADSC-CM treatment increased the beclin-1 levels, further confirming that the overexpression of this autophagy regulator can reduce α-syn aggregation and neurodegeneration in PD [81]. High levels of mTOR may also lead to an increase in beclin-1 and an associated decrease in oxidative stress [82].

Autophagy is carried out by a multistage process known as the autophagic flux, which can either induce cell death or protect cells against apoptosis. Autophagy involves the engulfment of bulk cytosolic material within autophagosomes, which then fuse with lysosomes wherein various enzymes reside to degrade the sequestered proteins. Therefore, the system plays an important role in maintaining the homeostasis of neuronal cells. The formation of LC3B-II from LC3B-I is related to the maturation of autophagosomes [23]. Upon recognizing ubiquitinated and aggregated proteins, p62 interacts with LC3B to initiate autophagosome formation for the degradation of those proteins in the lysosomes [24]. In this study, ROT caused excessive LC3B-II activation in both the Triton X-100-soluble and -insoluble fractions, suggesting an increase in the number of autophagosomes formed in neurons with NDD [83]. We also found accumulation of the insoluble p62 in the ROT-exposed SH-SY5Y cells. ROT enhanced the AMPK-dependent induction of both the LC3B-II and p62 levels, indicating that it blocks the autophagic flux for protein degradation [57,84]. The increases in insoluble α-syn, LC3B-II form, and p62 levels tend to increase insoluble cytosolic protein aggregates such as Lewy bodies and Lewy neurites, which impair autophagic flux and can result in neuronal cell death [85,86]. The attenuation of LC3B-II formation by NI-hADSC-CM provides evidence of the beneficial role of this therapy in autophagy-related toxicity. Additionally, the activation of mTOR can inhibit autophagy by degrading DEPTOR [87]. However, the reason for the increased p62 levels in both the Triton X-100-soluble and -insoluble fractions during NI-hADSC-CM treatment requires further study. Because its Akt-activated overexpression induces neuronal differentiation and cell survival [88], p62 may be involved in various regulatory mechanisms and attenuate the onset of apoptosis, suggesting that alternative pathways may exist.

The ATG family of proteins is involved in the formation and maturation of autophagosomes during autophagy [35], and disruption of its expression in neurons causes neuronal degeneration [89]. LC3B-I requires the E1-like enzyme ATG7 and the E2-like enzyme ATG3 to be converted into LC3B-II [90]. The conjugation of ATG5 and ATG12 elongates the phagophore membrane to form mature autophagosomes, which LC3B-II is a specific marker of [35]. In this study, the low levels of ATG3, 5, 7, and 12 during ROT-induced toxicity suggest that basal autophagy was suppressed, resulting in the marked accumulation of p62 in both the detergent-soluble and -insoluble fractions [6,89]. As expected, NI-hADSC-CM treatment induced an increase in the ATG protein levels, which coincided with increased mTOR levels [82], for survival of the excitatory neurons in this study.

Because of the protein degradation and amino acid recycling processes that occur within them, lysosomes serve as depots for intracellular nutrients [91]. Any deficit in lysosomal functions leads to the pathological accumulation of autophagosomes and inhibition of the autophagic flux, thereby also preventing the incorporation of p62 into those compartments for successful clearance [92]. In this study, ROT decreased the levels of the lysosomal proteins LAMP1, LAMP2, and TFEB in SH-SY5Y cells. As lysosomes are easy targets of oxidative stress, the decrease in lysosomal protein levels in PD concomitantly lead to the accumulation of α-syn, resulting in its failed degradation in autolysosomes [93]. LAMP1 deficiency has been reported in PD patients [94] and in α-syn-overexpressing cells [95] and is associated with increased LC3B-II levels [94,96]. LAMP2 deficiency has been implicated in the impaired fusion between misfolded α-syn-containing autophagosomes and lysosomes [83]. In this study, the ROT-induced inactivation of mTOR promoted autophagy by altering lysosomal biogenesis through the downregulation of TFEB expression. The increase in the levels of lysosomal proteins mediated by NI-hADSC-CM treatment may be due to the redistribution of lysosomes. The activation or overexpression of LAMP1, LAMP2, and TFEB has been shown to improve the clearance of aggregated α-syn [7,46]. Another study concluded that mTORC1 activation was required for its recruitment to the lysosomal surface and the subsequent activation of other regulatory molecules [91]. The results in this study further support the significant involvement of the lysosomal proteins in ROT-induced toxicity.

Previously, it has been described that the protein profile secreted by MSCs could induce a neuroprotective effect [97]. The complete autophagy protein profile analysis of the secretome in this present study showed that NI-hADSC-CM displayed higher expression levels of 20 autophagy-related proteins than hADSC-CM. Therefore, the stimulatory effect of NI-hADSC-CM was due to the influence of its secreted proteins, which may be crucial for developing a cell-free therapeutic option in the treatment of neurodegenerative diseases.

## 4. Materials and Methods

### 4.1. Conditioned Medium Collection from hADSCs and NI-hADSCs

Adipose tissues were obtained from individuals according to the guidelines established by the Ethics Committee at the Chonnam National University Medical School (IRB: I-2009-03-016). hADSCs were cultured and differentiated into NI-hADSCs using the methods established in our previous studies [31,36,37]. The hADSCs were grown as adherent cultures in Dulbecco’s modified Eagle’s medium (DMEM; Hyclone, Logan, UT, USA) supplemented with 10% FBS (Hyclone), 1% penicillin–streptomycin (Gibco BRL, Grand Island, NY, USA), and 0.2% amphotericin B (Gibco) at 37 °C in a humidified atmosphere containing 5% CO_2_. For the experiment, hADSCs (passages 3–5) were maintained in DMEM containing 1% FBS for 7 days, following which the cell culture medium (hADSC-CM) was aspirated, pooled, sterile filtered using a 0.2 μm syringe filter, and stored at −80 °C until further use. To induce neurogenic differentiation, hADSCs (passages 3–5) were maintained in DMEM containing 1% FBS and supplemented with 100 ng/mL bFGF (Invitrogen Co., Carlsbad, CA, USA) for 7 days. The cells were then incubated in the presence of 10 μM forskolin (Sigma Chemical Co., St. Louis, MO, USA) for the next 7 days. Then, the neural-induced conditioned medium (NI-hADSC-CM) was aspirated, pooled, sterile filtered using a 0.2 μm syringe filter, and stored at −80 °C until further use. Multiple batches of hADSC-CM and NI-hADSC-CM were collected for the subsequent experiments.

### 4.2. SH-SY5Y Cell Culture

The human neuroblastoma cell line SH-SY5Y (RRID: CVCL_0019; ATCC^®^ CRL-2266; American Type Culture Collection, Manassas, VA, USA) was maintained in DMEM (Welgene Inc., Gyeongsan, Korea) supplemented with 10% FBS and 1% penicillin–streptomycin at 37 °C in a humidified atmosphere containing 5% CO_2_/95% air. Confluent cultures (passages 15–22) were washed with phosphate-buffered saline (PBS), detached with 0.25% trypsin–EDTA solution, reseeded at a density of 50,000 cells/mL in DMEM containing 1% FBS, and used for the experiments after overnight incubation.

### 4.3. Rotenone Preparation

A 10 mM stock solution of ROT (R8875, Sigma) was prepared in dimethyl sulfoxide (DMSO; D2650, Sigma), aliquoted, stored at −80 °C, and used within 6 months. Before starting each experiment, a ROT working solution was prepared by diluting the stock solution with serum-free DMEM.

### 4.4. ROT Toxicity and hADSC-CM and NI-hADSC-CM Treatments

Time-course experiments were performed to characterize the protein signaling pathway changes mediated by the ROT-induced toxicity (a schematic of the experimental plan is depicted in Supplementary Figure S1a). At various timepoints of ROT exposure, the floating cells in the culture medium were pelleted by centrifugation, while the adherent cells were harvested by scraping them off the culture plate and then pelleting them by centrifugation. The two pellets were combined and washed twice with PBS, following which cell lysates were prepared for the Western blot analysis of target proteins.

To test the therapeutic effects of NI-hADSC-CM, SH-SY5Y cells were first incubated in the presence or absence of 0.5 μM ROT for 24 h. Then, after removing the culture medium, the floating cells were pelleted by centrifugation and resuspended in fresh medium and added to the wells of a culture plate. Either hADSC-CM or NI-hADSC-CM (each diluted at 50% in DMEM supplemented with 1% FBS) was added to the designated wells, and all cells (including the untreated ones) were incubated in the absence or presence of 0.5 μM ROT for another 24 h. FBS was maintained at a concentration of 1% throughout the study. Multiple sets of experiments were performed.

### 4.5. Reactive Oxygen Species Estimation

The intracellular ROS level was measured using a nonfluorescent dye, 2′,7′,-dichlorofluorescin diacetate (H_2_DCFDA; D6883, Sigma), which is a membrane-permeable fluorogenic tracer that is oxidized by various ROS. H_2_DCFDA (100 μM) was added to the cells of the various groups at 46 h of the experiment. At the end of the experiment at 48 h, the floating cells in the medium were combined with the trypsinized adherent cells, washed twice with ice-cold PBS, and suspended in warm PBS. The fluorescence intensity in each well of the black plastic microplate was measured at an excitation wavelength of 485 nm and emission wavelength of 538 nm using a fluorescence microplate reader (SpectraMax M2, Molecular Devices, Sunnyvale, CA, USA) and quantitated with SoftMax Pro 5.4.6 software (Molecular Devices). The values were calculated as the relative fluorescence intensity compared with that of the control group and expressed as a percentage (%) of the control. The assay was performed in triplicate.

### 4.6. Preparation of Triton X-100-Soluble and -Insoluble Fractions and Western Blotting of Target Proteins

Different passages of SH-SY5Y cells were treated with different batches of hADSC-CM or NI-hADSC-CM, following which Triton X-100-soluble and -insoluble (2% SDS soluble) cell lysate fractions were prepared for the Western blot analysis of target proteins. In brief, SH-SY5Y cells were incubated in the absence or presence of 0.5 μM ROT or the solvent (DMSO) for 24 h. After removing the medium, hADSC-CM or NI-hADSC-CM (at 50% dilution in DMEM) was added to the cells designated for the specific treatment, and all cells were then incubated in the presence or absence of 0.5 μM ROT for another 24 h (a schematic of the experimental plan is depicted in Supplementary Figure S1b). After 48 h, the floating cells in the medium were combined with the adherent cells (the latter harvested by scraping and centrifugation) and washed twice with PBS. Then, the cells were exposed to Triton X-100-soluble cell lysate buffer (100 mM Tris-HCl (pH 7.6), 100 mM NaCl, 1% Nonidet P-40, 1% sodium deoxycholate, 0.1% SDS, and 1% Triton X-100) supplemented with protease and phosphatase inhibitors and incubated for 30 min on ice. Thereafter, the cells were centrifuged at 13,200 rpm for 15 min at 4 °C, and the supernatants were collected as the Triton X-100-soluble fractions. The cell pellets were washed with PBS, dissolved in a Triton X-100-insoluble cell lysis buffer (containing protease and phosphatase inhibitors with 1% Triton X-100 and 2% SDS), and sonicated six times on ice for 10 s each time. The protein concentrations were determined using the BCA Protein Assay Kit (#23225, Thermo Scientific, Rockford, IL, USA) according to the manufacturer’s instructions. Thereafter, equal amounts of the proteins (10 or 15 μg) were separated on 6–12% SDS–polyacrylamide gels, following which the protein bands were transferred onto polyvinylidene difluoride membranes (IPVH00010, Millipore, Bradford, MA, USA).

The membranes were washed with Tris-buffered saline (TBS) containing 0.5% (*v*/*v*) Tween 20 (TBS-T), then blocked with a blocking buffer (5% nonfat dried milk solution or 1% bovine serum albumin (BSA) solution) prepared in TBS-T and subsequently incubated overnight with primary antibodies at 4 °C. The antibodies used (acquired from Abcam, Cambridge, MA, USA; Cell Signaling Technology Inc., Danvers, MA, USA; and Santa Cruz Biotechnology, Santa Cruz, CA, USA) are listed in Supplementary Table S1. Thereafter, the membranes were exposed to horseradish peroxidase-conjugated secondary antibodies for 2–3 h at ambient temperature and then washed three times with TBS-T. Finally, the signals were detected using an enhanced chemiluminescence (ECL) system (WBLUR0500, Millipore, Billerica, MA, USA) and a luminescent image analyzer (LAS 4000, GE Healthcare, Little Chalfont, UK). After visualizing the phosphoprotein forms, the membranes were washed with TBS-T and incubated in Western Blot Stripping Buffer (#21059, Thermo Scientific) with constant shaking for 60 min. Thereafter, the membranes were washed three times with TBS-T for 10 min each time, then blocked in TBS-T for 60 min and subsequently incubated overnight at 4 °C with the primary antibodies against the total-protein forms diluted in blocking buffer. Then, the membranes were washed three times with TBS for 10 min each time and incubated with the secondary antibodies diluted in blocking buffer. Finally, the membranes were washed three times in TBS for 10 min each time, and the signals were visualized using the ECL system. Beta-actin or GAPDH was used to normalize the expression levels of the target proteins. Densitometric analysis was performed using ImageJ software (National Institutes of Health, Bethesda, MD, USA).

### 4.7. Secretome Analysis by Autophagy Array Kit

A Human Autophagy Array Kit (AAH-ATG-1-8, RayBiotech^®^, Atlanta, GA, USA) was used. The expression levels of 20 human proteins related to autophagy were semiquantitatively determined for hADSC-CM and NI-hADSC-CM according to the manufacturer’s instructions. Briefly, each membrane was incubated with 1.5 mL of conditioned medium with specific antibodies precoated on the membrane at 4 °C overnight and washed accordingly. The membranes were further incubated with biotinylated antibody cocktail for 2 h and then with HRP-conjugated streptavidin for 2 h at room temperature. The membranes were washed, and the signals were visualized using the ECL system and a luminescent image analyzer (LAS 4000) by taking serial pictures with an exposure time of 10 min. The intensity of spot pixel of each on the membrane was determined by ImageJ software with “microarray profile” plugins. The background intensity was subtracted, and the values were finally calculated as percentages. Values above 5% were classified as secreted, those above 20% were considered abundantly expressed, and those below 5% were ignored.

### 4.8. Statistical Analysis

All data are expressed as the mean ± standard error of the mean (SEM) of three independent experiments. Data processing was performed using Microsoft Excel, and the GraphPad Prism^®^ 5.0 software (GraphPad Software Inc., San Diego, CA, USA) was used for analyzing the data and plotting the graphs. The statistical significance of the treatment effects was determined using one-way analysis of variance followed by Tukey’s post hoc multiple-comparison test. Autophagy array was performed with two-way analysis of variance. Differences with *p* values of less than 0.05 were considered statistically significant.

## 5. Conclusions

**Figure 9:**
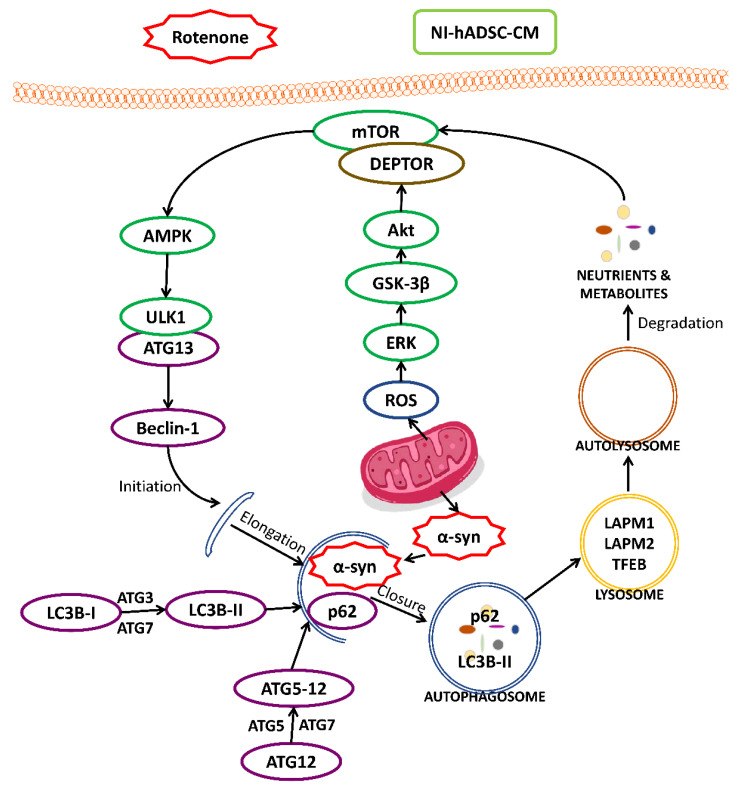
Diagrammatic representation of various activities of NI-hADSC-CM against ROT-induced toxicity in SH-SY5Y cells.

Autophagy involves several steps: namely, autophagy induction, membrane elongation, autophagosome formation, autophagosome–lysosome fusion, and target protein degradation. This autophagic flux is important for the removal of damaged mitochondria and aggregated α-syn, and excessive autophagic stress can contribute to neurodegeneration and cell death. In this study, ROT-induced toxicity increased the formation of ROS, which contributed to the deactivation of the mTOR pathway and triggered the formation of faulty autophagosomes, thereby inhibiting their incorporation of p62 and fusion with lysosomes for the degradation of their contents. The consequence of these defects was the prevention of the timely removal of dysfunctional mitochondria to protect cells from excessive autophagic and apoptotic stress. NI-hADSC-CM treatment reduced the production of ROS by mitochondrial complex I and restored the ROT-induced energy deficit in SH-SY5Y cells. The therapy led to the phosphorylation and activation of mTOR, which in turn led to the activation of ULK1, Akt, ERK, and ATG and lysosomal proteins, resulting in the inhibition of autophagy in vitro. Aside from its protective activities against oxidative stress- and autophagy-induced cell death, NI-hADSC-CM also attenuated the phosphorylation and oligomerization of α-syn during ROT-induced toxicity [4]. Taken together, these results indicate that NI-hADSC-CM exerts therapeutic effects through the inhibition of ROS-induced cell death, the stabilization of α-syn, and the suppression of autophagy and apoptosis (Figure 9). Moreover, the decreased cell numbers and increased ROS levels in control cells treated with hADSC-CM showed that NI-hADSC-CM had a higher therapeutic effect owing to the biological molecules released into the conditioned medium during neural differentiation of the hADSCs.

## Data Availability

Detailed raw data may be found in “Supplementary Materials” available with the online version of this article.

## Supplementary Materials

The following supporting information can be downloaded at: https://www.mdpi.com/article/10.3390/ijms23084193/s1.
