# Supplementary material for: Autophagy Signaling by Neural-Induced Human Adipose Tissue-Derived Stem Cell-Conditioned Medium during Rotenone-Induced Toxicity in SH-SY5Y Cells
Source: Int J Mol Sci. 2022 Apr 10;23(8):4193. doi: 10.3390/ijms23084193 (PMC9031864; doi:10.3390/ijms23084193)
Supplement: Supplementary file 1 [file ijms-23-04193-s001.zip › ijms-1672639-supplementary.pdf]

Supplementary materials to

Article

Autophagy Signaling by Neural-induced Human Adipose Tissue-derived Stem Cell-conditioned Medium during Rotenone-induced Toxicity in SH-SY5Y Cells

Mahesh Ramalingam [1,*,†], Han-Seong Jeong [1,†], Jinsu Hwang [1], Hyong-Ho Cho [2], Byeong C. Kim [3], Eungpil Kim [4] and Sujeong Jang [1,*]

1 Department of Physiology, Chonnam National University Medical School, Hwasun, Jeollanam-do 58128, Korea; jhsjeong@hanmail.net (H-S.J.); wlstn0128@naver.com (J.H.)
2 Department of Otolaryngology-Head and Neck Surgery, Chonnam National University Hospital, Chonnam National University Medical School, Gwangju 61469, Korea; victocho@jnu.ac.kr
3 Department of Neurology, Chonnam National University Hospital, Chonnam National University Medical School, Gwangju 61469, Korea; byeong.kim7@gmail.com
4 Jeonnam Biopharmaceutical Research Center, Hwasun, Jeollanam-do 58141, Korea; keungpil@gmail.com
* Correspondence: ramalingammahesh@jnu.ac.kr (M.R.); sujeong.jjang@gmail.com (S.J.)
† These authors contributed equally to this work.

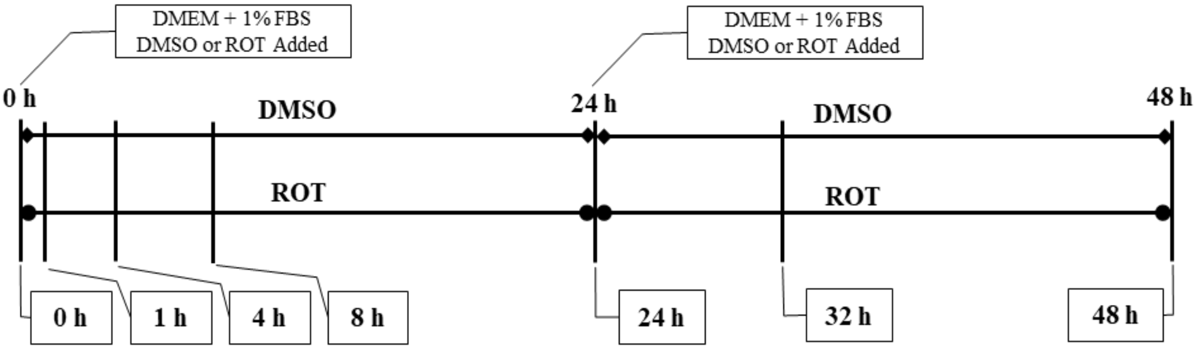

(a)

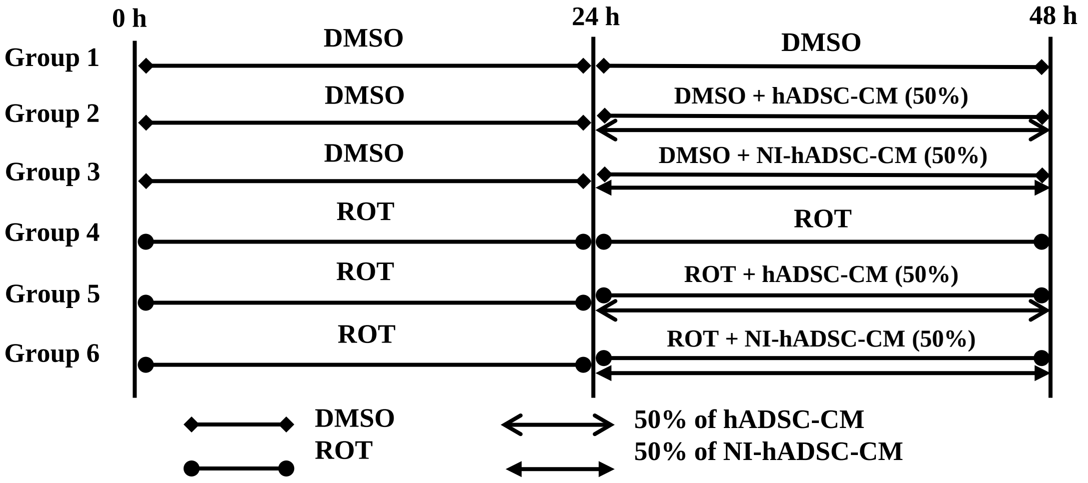

(b)

Supplementary Figure S1. (a) The experimental study plan for time course ROT toxicity. SH-SY5Y cells were seeded at a density of 50,000 cells/mL of DMEM containing 1% FBS and used for experiments after overnight incubation. Cells incubated in the absence or presence of ROT (0.5 μM) up to 48 h. Each time point, floating cells in medium were combined with adherent cells harvested by scraping, pelleted, and washed twice with PBS. Then, cells were used to prepare cell lysates. (b) To test the therapeutic effects of NI-hADSC-CM, SH-SY5Y cells were first treated with or without ROT for 24 h. The culture medium were removed, floating cells were pelleted from the medium, resuspend the cell pellet in the fresh medium, and added to respective wells. Then, cells were treated with or without hADSC-CM or NI-hADSC-CM at 50% dilution in DMEM supplemented with 1% FBS and incubated in the absence or presence of ROT (0.5 μM) for another 24 h. Floating cells in medium were combined with adherent cells harvested by scraping, pelleted, and washed twice with PBS. Then, cells were used to prepare cell lysates. Different passages of SH-SY5Y cells treated with different batches of hADSC-CM or NI-hADSC-CM for three independent experiments.

Supplementary Table S1. Western blotting conditions and antibodies used in this study.

Western blotting conditions:

SDS-PAGE Gel Percentages

- 6% = p-mTORC1, p-mTORC2, t-mTOR, p-ULK1, t-ULK1, ATG13, Beclin-1, p62, LAMP1, LAMP2, and TFEB
- 8% = p-AMPK and t-AMPK
- 9% = DEPTOR, p-Akt, t-Akt, p-GSK3β, t-GSK3, p-ERK, t-ERK, ATG5, ATG7, and ATG12
- 11% = LC3B, ATG3

SDS-PAGE Gel Running:

- 80~100 V for 100~120 min

SDS-PAGE Gel Transfer Times to PVDF Membrane:

- 6% = 250 mA for 90 min
- 8 & 9% = 220 mA for 90 min
- 11% = 200 mA for 60 min

Antibody Name	Host, MW Details	Company	Cat. No.	Dilution
**Primary Antibodies:**				
p-mTORC1 (Ser2448)	Mouse mAb, 220 kDa	Santa Cruz	sc-293133	1:500
p-mTORC2 (Ser2481)	Mouse mAb, 220 kDa	Santa Cruz	sc-293132	1:300
t-mTOR	Mouse mAb, 211~245 kDa	Santa Cruz	sc-517464	1:1,000
DEPTOR	Rabbit mAb, 48 kDa	Cell Signaling	#11816	1:1,000
p-AMPK (Thr172)	Rabbit mAb, 62 kDa	Cell Signaling	#2535	1:1,000
t-AMPK	Rabbit pAb, 62 kDa	Cell Signaling	#2532	1:1,000
p-ULK1 (Ser757)	Rabbit pAb, 140~150 kDa	Cell Signaling	#6888	1:1,000
t-ULK1	Rabbit mAb, 150 kDa	Cell Signaling	#8054	1:1,000
ATG13	Rabbit mAb, 72 kDa	Cell Signaling	#13273	1:1,000
p-Akt (Ser473)	Rabbit pAb, 60 kDa	Cell Signaling	#9271	1:1,000
t-Akt	Rabbit pAb, 60 kDa	Cell Signaling	#9272	1:2,000
p-GSK3β (Ser9)	Rabbit pAb, 46 kDa	Cell Signaling	#9336	1:1,000
t-GSK3	Rabbit mAb, 46 kDa	Cell Signaling	#9315	1:1,000
p-ERK1/2	Rabbit mAb, 44, 42 kDa	Cell Signaling	#4370	1:1,000
t-ERK1/2	Rabbit mAb, 44, 42 kDa	Cell Signaling	#4695	1:2,000
Beclin-1	Rabbit mAb, 60 kDa	Cell Signaling	#3495	1:1,000
LC3B	Rabbit mAb, 16,14 kDa	Cell Signaling	#3868	1:1,000
SQSTM1 = p62	Rabbit mAb, 62 kDa	Cell Signaling	#39749	1:1,000
ATG3	Mouse mAb, 42 kDa	Santa Cruz	sc-393660	1:500
ATG5	Mouse mAb, 32, 50 kDa	Santa Cruz	sc-133158	1:500
ATG7	Mouse mAb, 71 kDa	Santa Cruz	sc-376212	1:500
ATG12	Mouse mAb, 21, 60 kDa	Santa Cruz	sc-271688	1:500
LAMP1	Mouse mAb, 120 kDa	Santa Cruz	sc-20011	1:500
LAMP2	Rat mAb, 120 kDa	Santa Cruz	sc-20004	1:500
TFEB	Goat pAb, 53 kDa	Abcam	ab2636	1:1,000
GAPDH	Rabbit mAb, 37 kDa	Cell Signaling	#2118	1:3,000
GAPDH (HRP conjugate)	Rabbit mAb, 37 kDa	Cell Signaling	#8884	1:3,000
β-actin (HRP conjugate)	Rabbit mAB, 45 kDa	Cell Signaling	#5125	1:3,000
**Secondary Antibodies:**				
Anti-rabbit IgG, HRP-linked antibody		Cell Signaling	#7074	1:1,000
Anti-mouse IgG, HRP-linked antibody		Cell Signaling	#7076	1:1,000
Anti-rat IgG, HRP-linked antibody		Cell Signaling	#7077	1:1,000
Anti-goat IgG-HRP		Santa Cruz	sc-2354	1:1,000

p-, phosphorylated; t-, total.
pAb, polyclonal antibody; mAb, monoclonal antibody; kDa, kiloDalton.

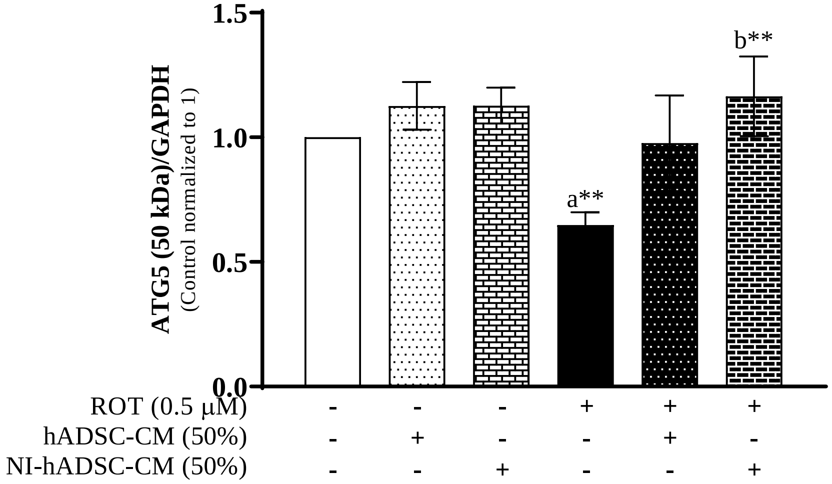

(a)

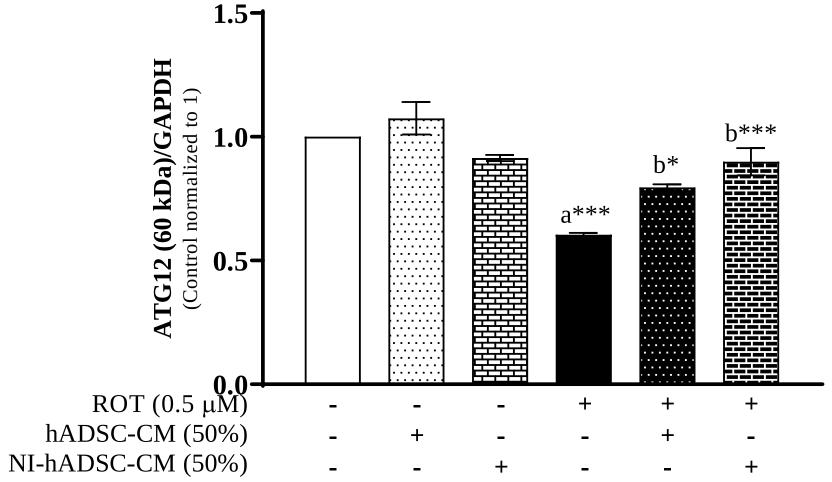

(b)

Supplementary Figure S2. SH-SY5Y cells were seeded as 50,000 cells/mL of DMEM containing 1% FBS and used for experiments after overnight incubation. Cells with absence or presence of ROT (0.5 μM) for 48 h were treated with hADSC-CM (50%) or NI-hADSC-CM (50%) during the last 24 h and analyzed by Western blotting. The bar graphs represents for ATG5-ATG12/GAPDH ratio (a) and ATG12-ATG5/GAPDH ratio (b). Data are mean ± SEM of three independent experiments and analyzed by one-way of variance (ANOVA) followed by Tukey's *post hoc* test. Statistical significance: a–compared with control; b–compared with ROT; * $p < 0.05$, ** $p < 0.01$ and *** $p < 0.001$.

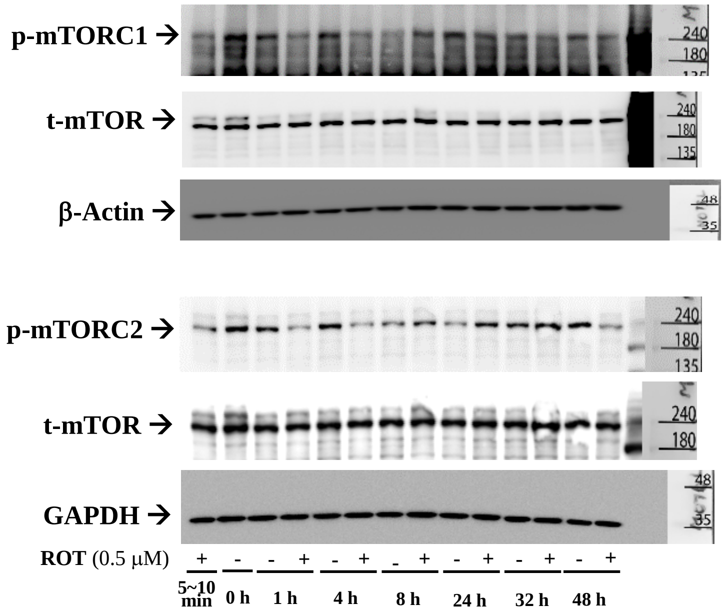

(a)

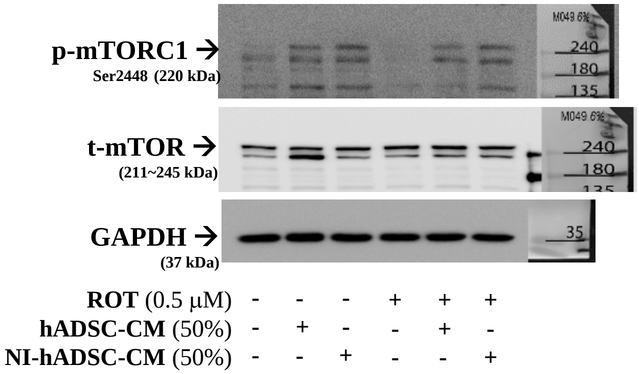

(b)

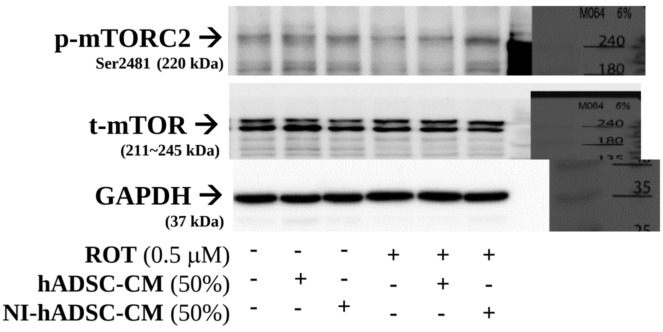

(c)

Supplementary Figure S3. Unedited images and their molecular weight markers for respective Western blots used in **Figure 2** of this manuscript.

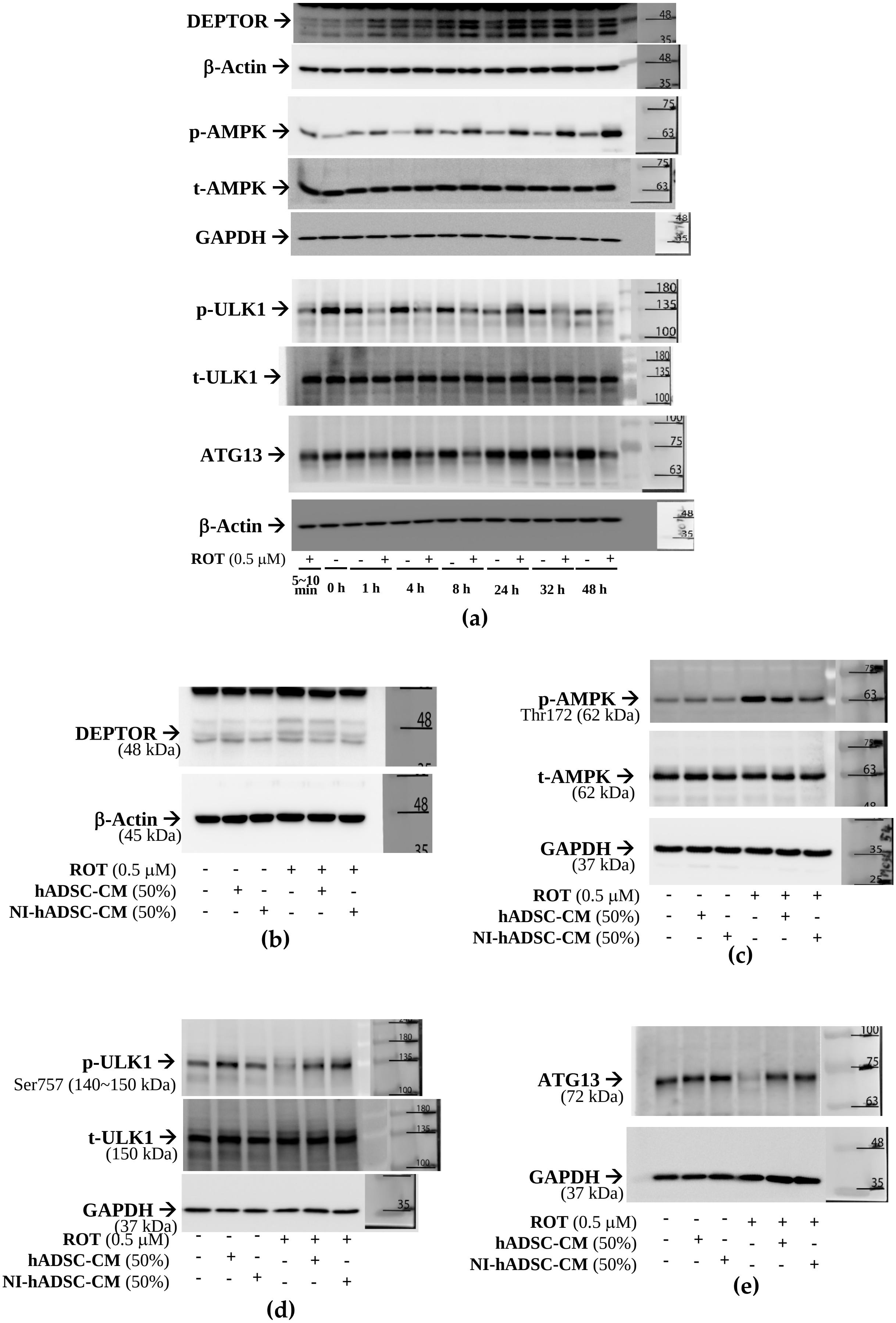

Supplementary Figure S4. Unedited images and their molecular weight markers for respective Western blots used in **Figure 3** of this manuscript.

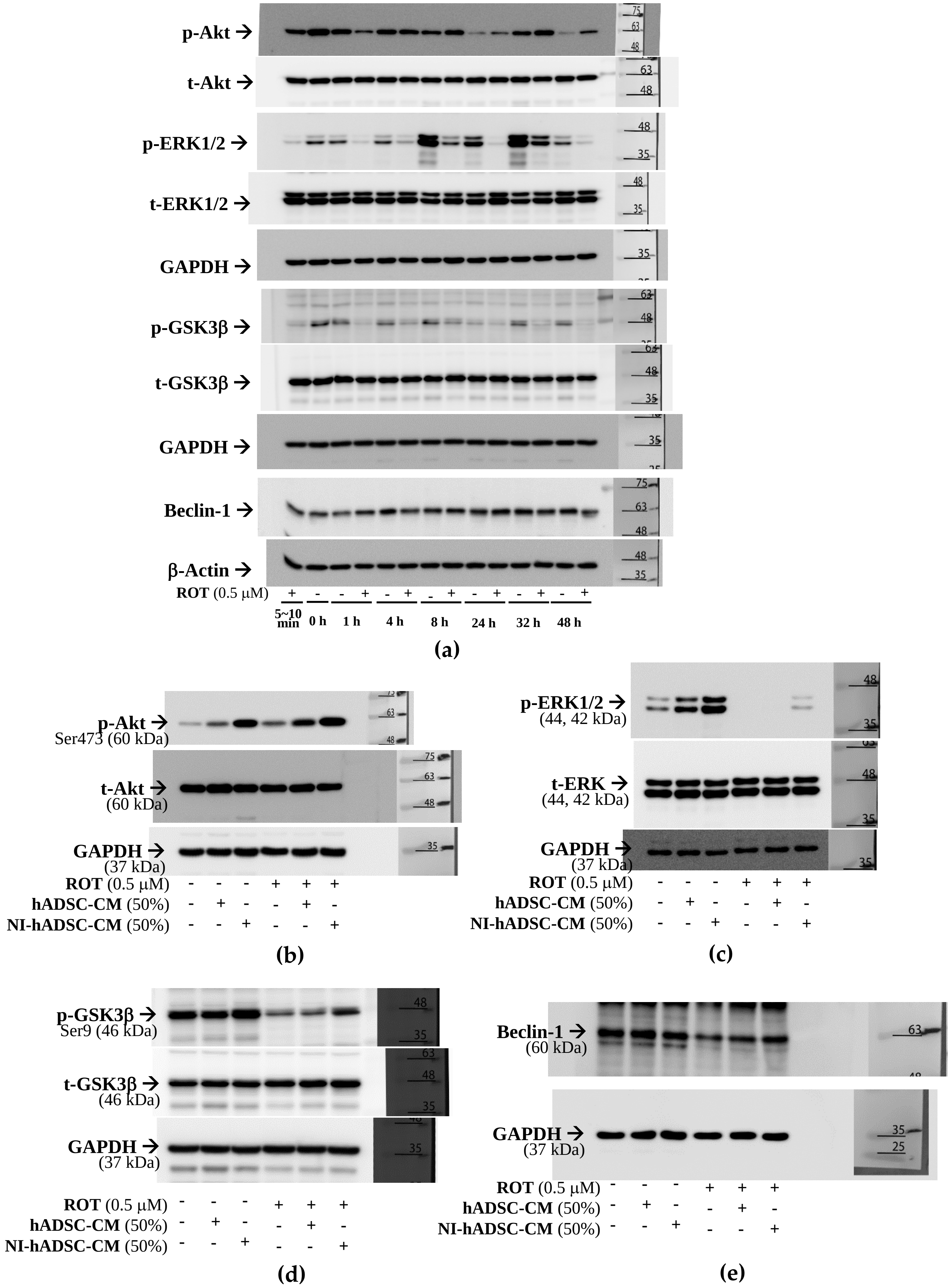

Supplementary Figure S5. Unedited images and their molecular weight markers for respective Western blots used in **Figure 4** of this manuscript.

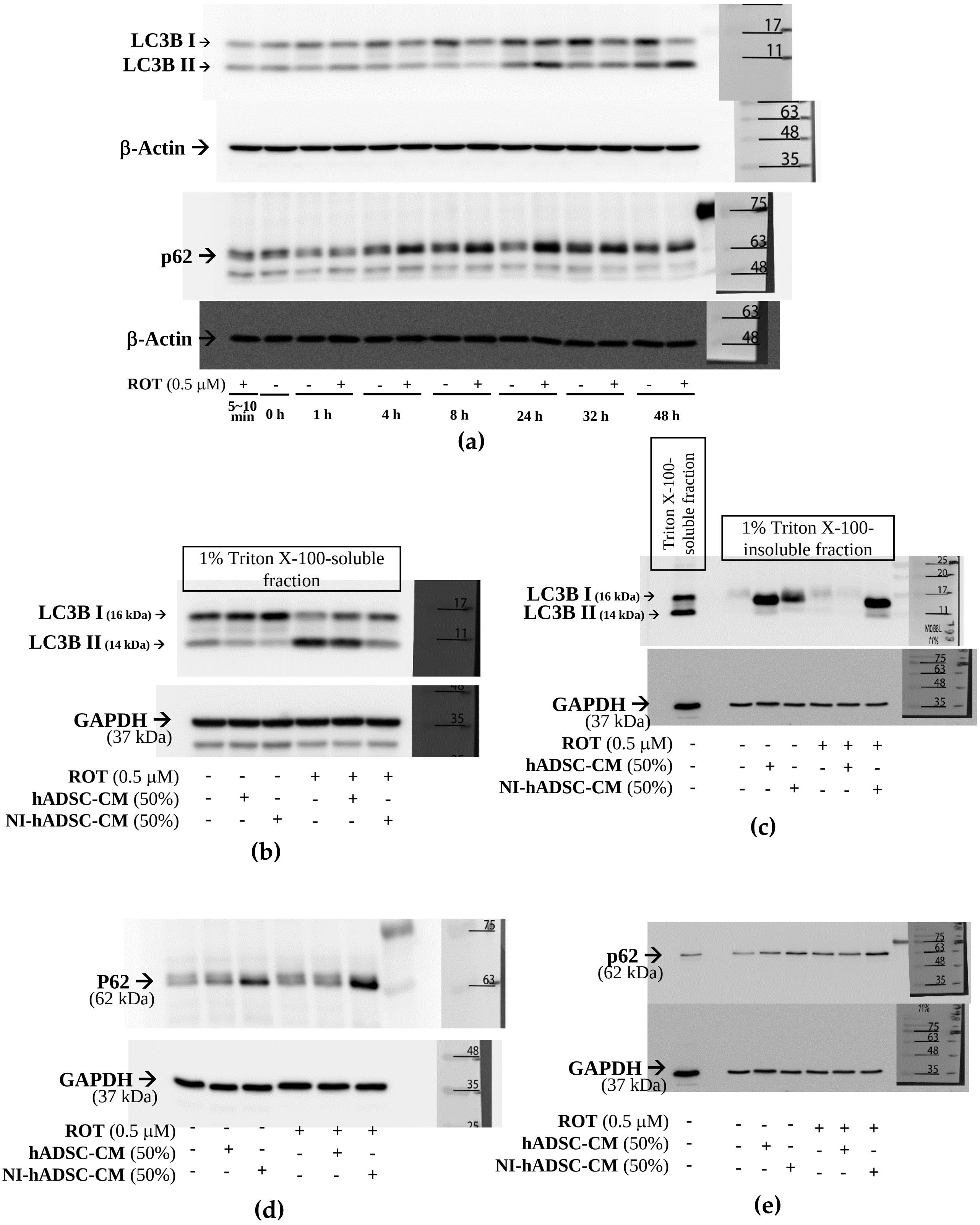

Supplementary Figure S6. Unedited images and their molecular weight markers for respective Western blots used in **Figure 5** of this manuscript.

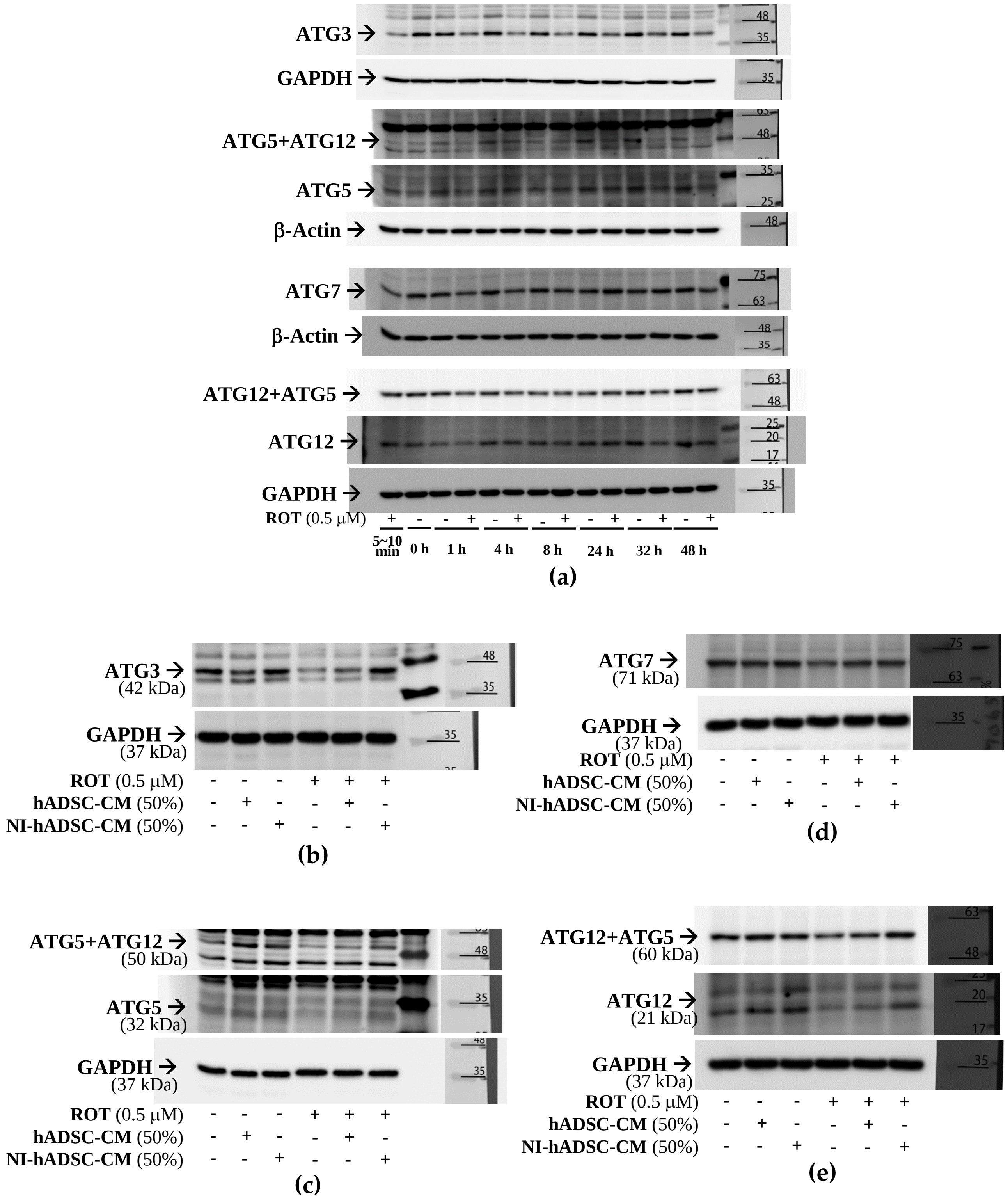

Supplementary Figure S7. Unedited images and their molecular weight markers for respective Western blots used in **Figure 6** of this manuscript.

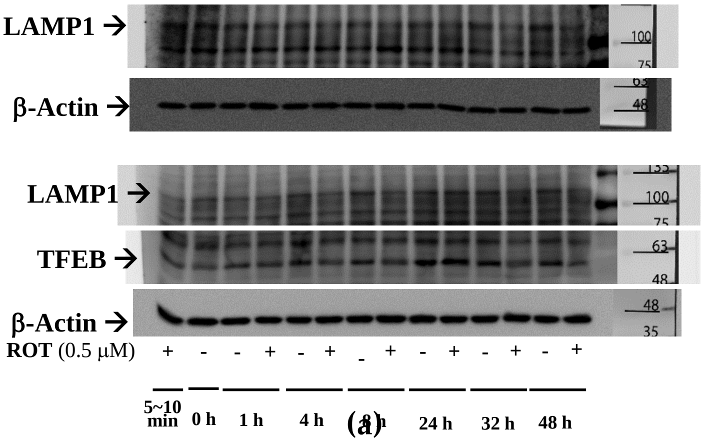

(a)

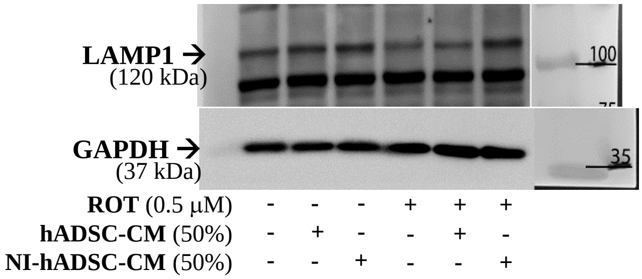

(b)

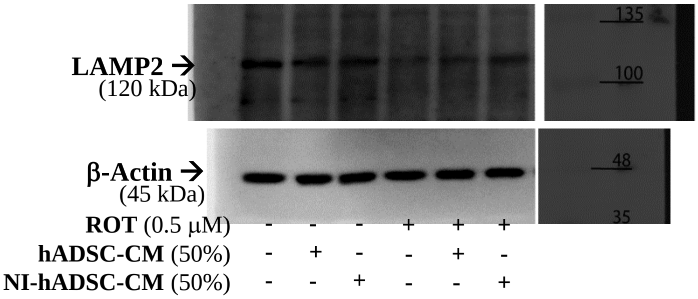

(c)

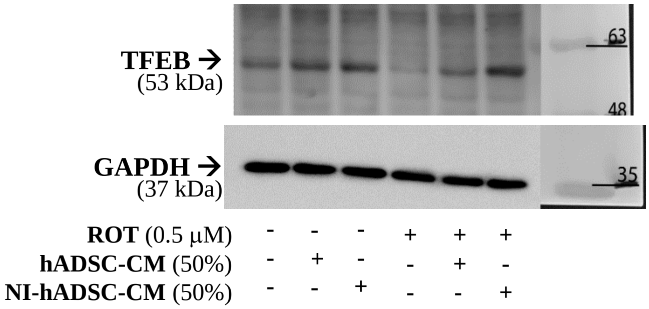

(d)

Supplementary Figure S8. Unedited images and their molecular weight markers for respective Western blots used in **Figure 7** of this manuscript.

Each antibody is spotted in duplicate vertically

	A	B	C	D	E	F	G	H
1<br>2	POS	POS	NEG	NEG	ATG3	ATG4A	ATG4B	ATG5
3<br>4	NEG	NEG	ATG7	ATG10	ATG12	ATG13	Beclin-1	BNIP3L
5<br>6	DDR2	GABARAP	LAMP1	LC3A	LC3B	NBS1	p62	NEG
7<br>8	Rheb	MSK1	α-syn	NEG	NEG	NEG	NEG	POS

(a)

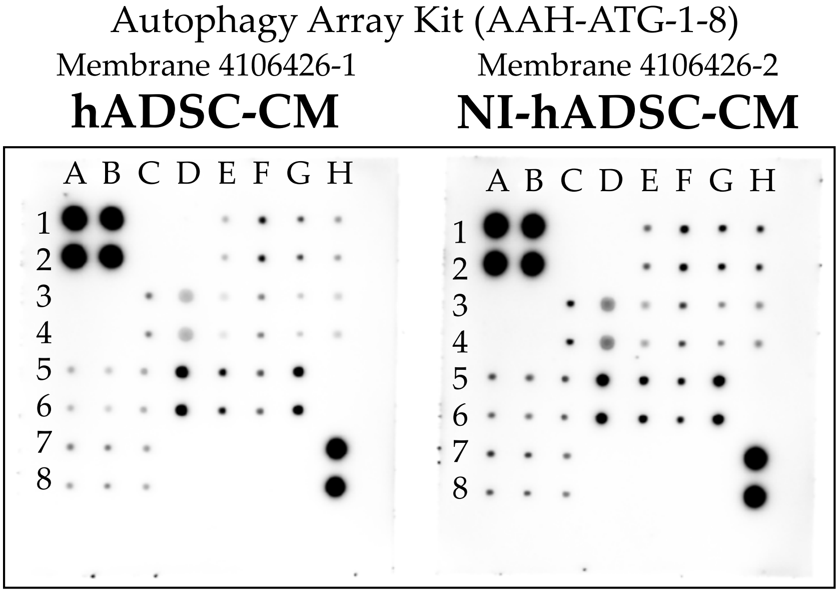

(b)

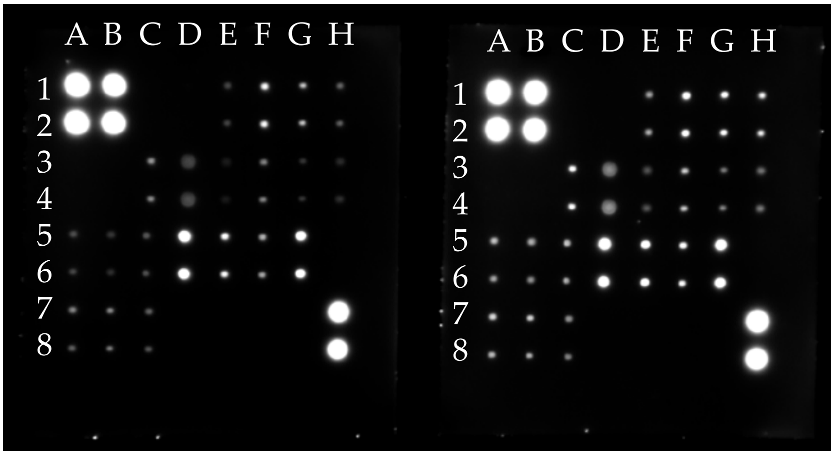

(c)

Supplementary Figure S9. (a) The human autophagy array (Raybiotech, AAH-ATG-1-8) profiles 20 autophagy related proteins spotted in duplicate, positive controls (POS), and negative controls (NEG). (b) Unedited image of autophagy array assay membranes for hADSC-CM and NI-hADSC-CM used in **Figure 8** of this manuscript. (c) Inverted image of (b) used for quantification in ImageJ software with 'Microarray Profile' plugins.

Supplementary Table S2. Quantification of the 20 proteins spotted on autophagy array membranes.

Analyte #	Coordinate	Analyte Name	Membrane 4106426-1, Sample: hADSC-CM		Membrane 4106426-2, Sample: NI-hADSC-CM		% Change of hADSC-CM vs. NI-hADSC-CM			>5 = Secreted >20 = Expressed	P Value Summary
			Replicate 1	Replicate 2	Replicate 1	Replicate 2	Replicate 1	Replicate 2	Mean		
**N/A**	1A, 2A	Positive Spots	64499.267	64503.903	64286.973	63551.897	-0.329	-1.476	-0.903	Positive Spots	N/A
**N/A**	1B, 2B	Positive Spots	62331.231	62614.114	63312.835	62493.567	1.575	-0.193	0.691	Positive Spots	N/A
**1**	1E, 2E	ATG3	2189.204	1761.440	4825.254	4617.007	120.411	162.115	141.263	Increased	NS
**2**	1F, 2F	ATG4A	7804.690	6959.292	10436.524	9086.105	33.721	30.561	32.141	Increased	NS
**3**	1G, 2G	ATG4B	4201.395	3902.102	8135.753	7091.378	93.644	81.732	87.688	Increased	*
**4**	1H, 2H	ATG5	2854.723	2285.914	7194.363	6444.486	152.016	181.922	166.969	Increased	**
**5**	3C, 4C	ATG7	4389.781	3507.000	8121.777	7400.483	85.016	111.020	98.018	Increased	**
**6**	3D, 4D	ATG10	6433.133	7420.976	12393.554	14002.530	92.652	88.689	90.670	Increased	***
**7**	3E, 4E	ATG12	1095.213	979.457	3078.256	3108.571	181.065	217.377	199.221	Increased	NS
**8**	3F, 4F	ATG13	3037.292	2838.953	5397.214	4767.254	77.698	67.923	72.811	Increased	NS
**9**	3G, 4G	Beclin-1	1094.736	1006.652	2919.784	2919.205	166.711	189.991	178.351	Increased	NS
**10**	3H, 4H	BNIP3L	1501.811	1626.546	4525.023	4758.469	201.304	192.551	196.927	Increased	*
**11**	5A, 6A	DDR2	3495.354	2714.383	6924.148	5130.357	98.096	89.006	93.551	Increased	NS
**12**	5B, 6B	GABARAP	1908.254	1435.079	6009.652	3917.148	214.929	172.957	193.943	Increased	*
**13**	5C, 6C	LAMP1	2574.797	2085.031	5870.840	4238.838	128.012	103.299	115.655	Increased	NS
**14**	5D, 6D	LC3A	22880.020	19838.790	25268.015	20985.037	10.437	5.778	8.107	Secreted	NS
**15**	5E, 6E	LC3B	8135.021	6809.913	12991.765	11633.998	59.702	70.839	65.270	Increased	***
**16**	5F, 6F	NBS1	4464.084	3793.830	8053.124	6620.057	80.398	74.495	77.447	Increased	*
**17**	5G, 6G	p62	16017.823	14392.930	21628.726	19132.166	35.029	32.928	33.978	Increased	***
**18**	7A, 7B	Rheb	4039.316	2332.128	6552.392	5237.571	62.215	124.583	93.399	Increased	NS
**19**	7B, 8B	MSK1	3168.930	2332.900	5174.946	3925.892	63.303	68.284	65.793	Increased	NS
**20**	7C, 7D	α-syn	2443.264	1797.604	3733.128	3002.337	52.793	67.019	59.906	Increased	NS

ATG, autophagy-related; BNIP3L, Bcl-2 interacting protein 3 like; DDR2, discoidin domain receptor tyrosine kinase 2; GABARAP, gamma-aminobutyric acid receptor-associated protein; LAMP1, lysosomal associated membrane protein 1; LC3A, microtubule associated protein 1 light chain 3 alpha; LC3B, microtubule associated protein 1 light chain 3 beta; NBS1, nibrin; p62, sequestosome 1; Rheb, Ras homology enriched in brain; MSK1, mitogen-and stress-activated protein kinase 1; α-syn, alpha-synuclein. Data collected by using ImageJ software with 'Microarray Profile' plugins. Mean (%) values above 5% were classified as secreted, above 20% were considered abundantly expressed, and below 5% were ignored. Statistical analysis was performed using two-way analysis of variance. Statistical significance: NI-hADSC-CM compared with hADSC-CM as control; * $p < 0.05$, ** $p < 0.01$, *** $p < 0.001$ and ^{NS}-non-significant.
